# Novel Spontaneous Nanoemulsions for Phosphorus Selenide Compounds: Toward Enhanced Solubility and Stable Aqueous Formulations

**DOI:** 10.1021/acsomega.5c08867

**Published:** 2025-11-11

**Authors:** Romelly Eugenia Rojas Ramírez, Daiani Canabarro Leite, Ana M. S. Recchi, Nadia Carollayn Correa da Silva, Yago Cezar Bastianello, Fabiola Caldeira dos Santos, Tielle Moraes de Almeida, Gilson Zeni

**Affiliations:** † Department of Biochemistry and Molecular Biology, UFSM, Santa Maria 97105-900, Brazil; ‡ Department of Physics, UFSM, Santa Maria 97105-900, Brazil

## Abstract

Organoselenium compounds have been extensively studied for their potential anticancer, hypoglycemic, antimicrobial, antioxidant, and anti-inflammatory properties. However, these compounds are highly lipophilic, restricts their solubility in organic solvents and limits their administration routes. Investigating surfactant interaction with organoselenium compounds offers opportunities to enhance formulation efficacy by increasing their bioavailability and water solubility through the formation of stable colloidal systems. In this study, phosphorus selenide compounds were mixed with three commonly used surfactants: cetyltrimethylammonium bromide (CTAB), sodium dodecyl sulfate (SDS), and Tween 20 (polysorbate 20). The resulting colloidal structures were characterized by ultraviolet–visible (UV–vis) spectroscopy, dynamic light scattering (DLS), ζ-potential (ZP), Fourier transform infrared (FTIR) spectroscopy, nuclear magnetic resonance (NMR) spectroscopy, and atomic force microscopy (AFM). Improved apparent solubility was observed for all compounds, with aliphatic phosphorus selenide compounds exhibiting greater compatibility with the surfactants than aromatic variants. Formulations with SDS and CTAB were more stable than those with Tween 20, likely due to unfavorable electrostatic interactions between the nonionic surfactant and the phosphorus selenide compounds. Particle sizes ranging from 100 to 250 nm were measured and, together with conductivity and AFM analyses, indicated the formation of spontaneous nanoemulsions. Studying surfactant interactions with these compounds may improve formulations by reducing the required amount of active compound required, enabling more precise release. This approach also reduces reliance on organic solvents, thereby minimizing environmental impact and offering promising applications in diverse fields, including pharmaceuticals, pesticide formulations, and catalysis.

## Graphical abstract

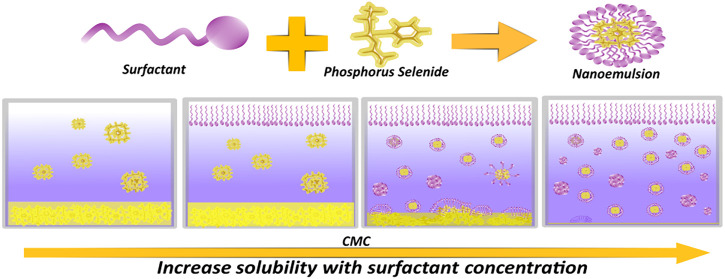

## 1 Introduction

Because of the potential role of colloidal systems, such as nanocarriers,^1–3^ the interaction between chalcogen organic compounds (including organoselenium compounds) and surfactants has garnered significant attention in various fields of research, particularly in biological and pharmaceutical applications. Micelles, nanoemulsions, liposomes, and others are formed when surfactant molecules self-assemble into structures with a hydrophobic core and a hydrophilic shell, enabling the solubilization, dispersion, transportation, and delivery of poorly water-soluble formulations.^4–7^

Self-assembled structures are crucial in drug delivery because they enhance the solubility and bioavailability of hydrophobic drugs, thereby improving their therapeutic efficacy. By encapsulating poorly water-soluble drug molecules within the hydrophobic core, these self-assembly systems can protect them from degradation and facilitate their targeted delivery to specific tissues or cells.^8–10^ This controlled release mechanism offers potential advantages in reducing drug toxicity and enhancing treatment efficiency in various diseases.

Spontaneous nanoemulsions are colloidal systems of interest because they form in a thermodynamically driven or kinetically favored manner, when an oil phase containing surfactants and, in some cases, cosurfactants is mixed with an aqueous phase under appropriate conditions of composition, temperature, and mild agitation, producing droplets typically in the size range of 100–600 nm.^11–14^

As an essential micronutrient, selenium plays a vital role in maintaining cellular function and overall health. Organoselenium compounds have been extensively studied for their potential anticancer, hypoglycemic, antimicrobial, antioxidant, and anti-inflammatory properties, underscoring their therapeutic promise in the treatment of various diseases.^15–18^ Phosphorus selenide compounds belong to this class of bioactive molecules and have been reported to exhibit enhanced pharmaceutical properties, particularly when incorporated into biological frameworks.^19,20^ Moreover, the development of novel phosphorus selenium derivatives holds significant potential to enhance the biological activity and pharmacokinetic profiles of selenium-based drugs, thereby paving the way for innovative therapeutic strategies.^15,16^

A key characteristic of organoselenium compounds is their high lipophilicity, which restricts their solubility predominantly to organic solvents or oils, such as dimethyl sulfoxide and vegetable oils, including canola and soybean, or mineral oils.^15^ This property limits the available administration routes. For example, Ebselen and its analogs,^20,21^ organoselenium compounds recognized for their antioxidant and anti-inflammatory properties, have been tested in clinical trials for their potential use in treating acute ischemic stroke. Ebselen was administered orally as a bulk suspension in water, which is, in turn, considered a nonoptimized dosage form.^22,22–25^

Furthermore, new applications in agriculture have been developed with organoselenium compounds as self-sealants, catalysts, pesticides, and herbicides.^26–29^ Interestingly, using surfactants as adjuvants in pesticides formulation increases their specificity and reduces their impact on the soil.^30–32^ Phosphorus selenide compounds combined with surfactants may enhance wettability and penetration, making them promising candidates for agrochemical applications.

The study about the interactions of surfactants with phosphorus selenide compounds can enhance formulations, improving their bioavailability and solubility in water, reducing the amount of compound needed, avoiding the use of organic solvents, and allowing more specific release, thereby minimizing environmental impact. The interaction of organoselenium compounds with surfactants and the exploration of new phosphorus selenide compounds represent exciting avenues for advancing biological applications and different technologies. The work by Mehta et al. shows the interactions of some surfactants with organoselenide compounds and the docking mechanism of surfactants and solubilizer molecules within the system.^4,5,33^ Also, Pawar et al. achieved better solubility in neutral surfactants with diphenyl diselenide compounds. In the case of these newly proposed phosphorus selenide compounds, no studies have been conducted with surfactants to improve solubility or dispersion in water and to enhance pharmacological properties.

In this work, mixtures of phosphorus selenide compounds, with three surfactants were studied: cetyltrimethylammonium bromide (CTAB), sodium dodecyl sulfate (SDS), and Tween 20 (polysorbate 20); these surfactants play a crucial role in stabilizing dispersed systems by reducing interfacial tension and enabling the formation of micelles, emulsions, and other colloidal structures.^34–36^ These three surfactants, representing cationic, anionic, and nonionic types, were deliberately selected to provide a broader understanding of the interactions between surfactants and phosphorus selenide compounds. These surfactants have been successfully employed to enhance the solubility of organoselenium compounds in water, thereby significantly improving their bioavailability.^37–39^ Although these surfactants are nontoxic at low concentrations and are therefore considered biocompatible, in sensitive biological contexts, they may cause toxicity depending on the dose.^40,41^

The solubility and dispersion of these compounds in surfactant aqueous systems were studied using UV–vis spectroscopy. The influence of these compounds on the critical micelle concentration (CMC) or critical aggregation concentration (CAC) of surfactants was determined by conductimetry. Additionally, the systems were characterized by using dynamic light scattering (DLS), ζ-potential (PZ), Fourier transform infrared spectroscopy (FTIR), nuclear magnetic resonance (NMR) spectroscopy, and atomic force microscopy (AFM).

## 2 Experimental Section

### 2.1 Materials

The phosphorus selenide compounds (R-PPhSe): diethyl Se-(4-methoxyphenyl) phosphorus selenide (C1), diethyl Se*-p-*tolyl phosphorus selenide (C2), diethyl Se-phenyl phosphorus selenide (C3), diethyl Se-(3-(trifluoromethyl)­phenyl) phosphorus selenide (C4), tris­(2-(phenylselanyl)­ethyl) phosphate (C5), and Se-butyl-diethyl phosphorus selenide (C6) were synthesized by LASRAFTO (Santa Maria, Brazil) following a previously published methodology.^42^ The ^1^H NMR and ^13^C NMR spectral data fully agreed with the assigned structure. The chemical purity (99.9%) was determined by gas chromatography–mass spectrometry (GC–MS; Shimadzu QP2010PLUS GC/MS combination). Cetyltrimethylammonium bromide (CTAB), sodium dodecyl sulfate (SDS), and Tween 20 (polysorbate 20) were bought from Delaware (Porto Alegre, Brazil) (chemical purity 99.9%). The water was obtained from a Millipore Milli-Q system (USA) pH ≈ 7 with conductivity 0.5 μS cm^–1^ and is referred to in the manuscript as ultrapure water. All other reagents were obtained from Sigma-Aldrich (São Paulo, Brazil) if not otherwise indicated.

### 2.2 Phosphorus Selenide Compounds Solubility in Surfactant Systems

An excess concentration of the phosphorus selenide compounds (Figure 1) was mixed in different concentrations of CTAB, Tween 20, and SDS (0–14 mmol L^–1^) overnight at 25 °C. The solutions were then centrifuged for 15 min at 2,500 rpm. The supernatant was analyzed using a UV–vis spectrophotometer (Shimadzu UV-1650PC, Japan) at the absorption wavelength of each compound, with a calibration curve prepared from pure samples.

**Figure 1:**
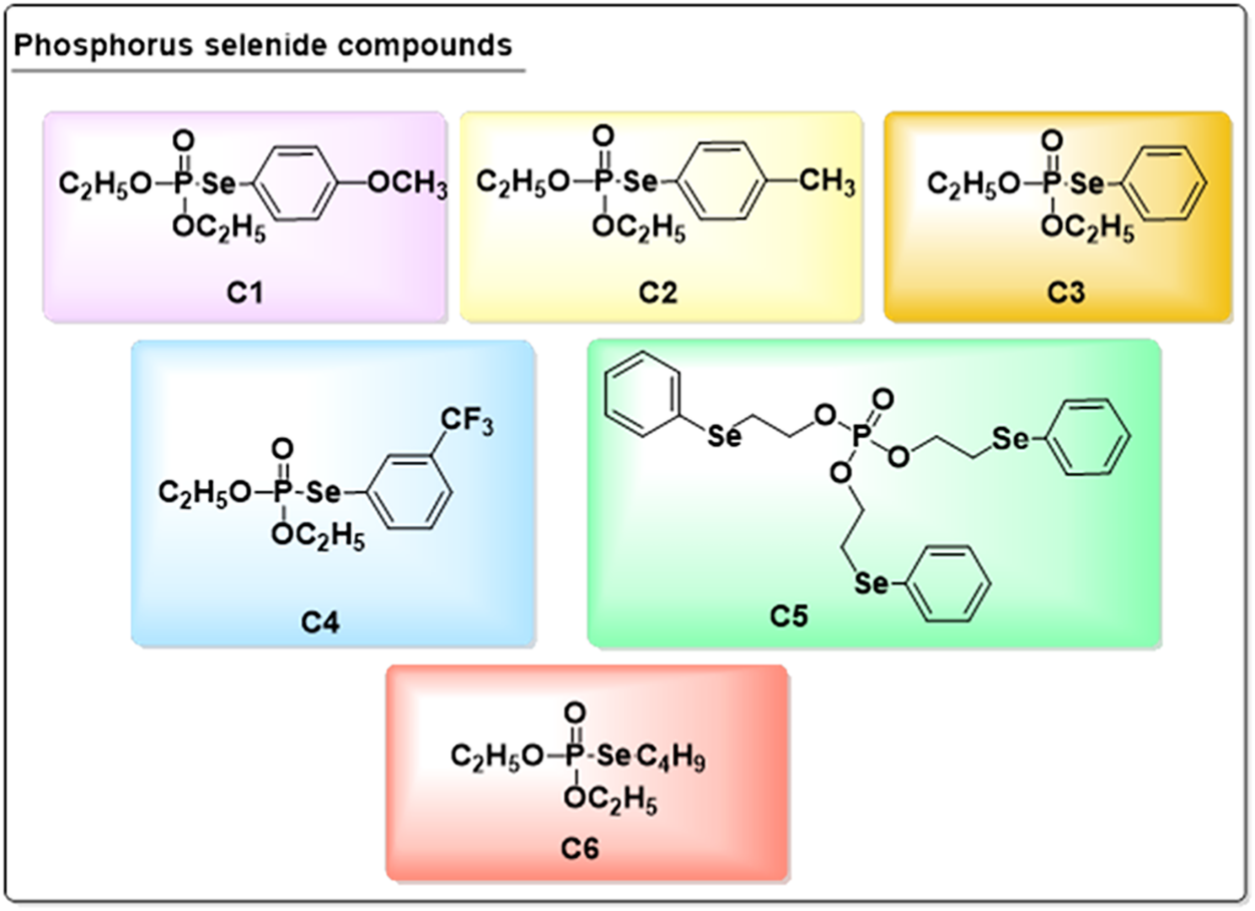
Structure and IUPAC nomenclature of phosphorus selenide compounds: diethyl Se-(4-methoxyphenyl) phosphorus selenide (C1), diethyl Se*-p-*tolyl phosphorus selenide (C2), diethyl Se-phenyl phosphorus selenide (C3), diethyl Se-(3-(trifluoromethyl)­phenyl) phosphorus selenide (C4), tris­(2-(phenylselanyl)­ethyl) phosphate (C5), and Se-butyl-diethyl phosphorus selenide (C6).

### 2.3 Dynamic Light Scattering (DLS)

Dynamic light scattering (DLS) measurements were carried out at 25 °C using a Brookhaven (BI200 M goniometer with a BI9000AT digital correlator) with a He–Ne vertical polarized laser (λ = 632.8 nm) at a fixed scattering angle (θ = 90°), coupled with a thermal bath. Data were processed using CONTIN (for size distribution and correlation function) and the cumulant method (for polydispersity index, PDI).^43,44^ Size distributions were calculated by number, and the values are reported as apparent hydrodynamic diameter (*D*_happ_ ± sd, nm). The hydrodynamic diameter was obtained from DLS measurements using the Stokes–Einstein relation, with the viscosity of pure water at 25 °C assumed for all calculations. Given the dilute conditions investigated (1–14 mM), this approximation is considered appropriate, as the bulk viscosity of the solutions remains essentially unchanged relative to water.

### 2.4 ζ-Potential

The ζ-potential was determined by microelectrophoresis without dilution, using a Zetasizer Nano-ZS (model ZEN 3600) from Malvern Instruments (UK). Results were expressed as mean (mV) ± standard deviation of three samples. The instrument software employs the Smoluchowski approximation (*f*(κa) = 1.5), which is the most appropriate model under our experimental conditions. Values of ζ > 50 mV are considered apparent ζ-potentials.

### 2.5 Determination of CMC and CAC by Conductimetry and Thermodynamic Analysis of Aggregation

The critical micelle concentration (CMC) and critical aggregation concentration (CAC) values of SDS, CTAB, and their mixtures with the compounds were determined by conductometric measurements. The specific conductivity was measured using a digital conductivity meter (EC Meter, Brazil) with an absolute accuracy of 3% and a precision of 0.1%. A thermal bath maintained a temperature of 25 °C within 0.1 °C.

The global free energy of aggregation, Δ*G*°_agg_, can be interpreted as an approximation of the free energy of self-association in an aqueous medium (for larger aggregates, such as vesicles or emulsions), rather than the formation of an ideal spherical micelle. This is related to the CAC by the following equation

1 $${\Delta G^{{}^{\circ}}}_{\mathrm{agg}}=\left(2-\beta \right)\mathrm{RT}\ln X_{\mathrm{CAC}}$$

where *R* is the ideal gas constant (8.314 J K^–1^ mol^–1^), *T* is the absolute temperature (K), β is the surfactant degree of ionization, and *X*_CAC_ is the molar fraction.^33,46^

### 2.6 Fourier Transform Infrared Spectroscopy with Attenuated Total Reflectance (FTIR-ATR) Spectroscopy

FTIR-ATR analysis was conducted on a Vertex 70 (BRUKER, Germany) spectrophotometer. For each sample (with the surfactant or the pure compound), 100 scans were performed, with a 2 cm^–1^ resolution and a wavelength range of 30 to 4000 cm^–1^. Samples were freeze-dried before analysis (Micromodulo, ThermoElectron Corporation, USA, software OPUS).

### 2.7 Nuclear Magnetic Resonance (NMR) Spectroscopy

The ^1^H and ^13^C nuclear magnetic resonance spectra were obtained at 400 MHz (Bruker Avance III HD) and showed analytical and spectroscopic data in complete agreement with the assigned structure. Samples were freeze-dried before analyses (Micromodulo, ThermoElectron Corporation, USA, software OPUS).

The analyses of the surfactants CTAB, Tween 20, phosphorus selenide, and the phosphorus selenide-surfactant formulations were conducted by dissolving 25 mg of each compound in 0.5 mL of deuterated chloroform (CDCl_3_). To analyze the SDS, 25 mg was dissolved in 0.5 mL of deuterated dimethyl sulfoxide (DMSO-*d*_6_).

### 2.8 Atomic Force Microscopy (AFM)

The morphology of nanoemulsions was analyzed using atomic force microscopy (AFM). The samples for AFM were prepared by depositing sample C1_SDS at 14 mmol L^–1^ on freshly cleaved mica plates adhered to a polystyrene plate and then evaporated at 25 °C. Samples were scanned in noncontact modes using no-contact cantilever AC160 TS, with *k* equal 26 N m^–1^ and *f* approach 300 kHz. Topographic images were acquired using a Park NX10 (Park Systems, Suwon-Korea) microscope equipped with SmartScan software version 1.0. RTM11a. All measurements were performed at a temperature of 25 ± 3 °C and relative humidity of 55 ± 10%. The images were processed using XEI software version 4.3.4 Build22.RTM1 and ImageJ.

### 2.9 Statistical Analysis

Results were expressed as mean ± SD. One-way ANOVA with Bonferroni’s multiple comparison post hoc tests was performed to assess statistical significance using Graph Pad Prism 5.0 software. The comparison between the groups was considered significant if *p* < 0.05.

## 3 Results and Discussion

### 3.1 Solubility in Water: Mixture of Neutral, Anionic, and Cationic Surfactants with Phosphorus Selenide Compounds

The solubility was determined using UV–vis spectroscopy, employing calibration curves for each compound studied. Using surfactants enables the dispersion or apparent solubility of various lipophilic molecules in organic solvent-free formulations, thereby improving their aqueous bioavailability, vectorization, and delivery.

An increase in the solubility and dispersion of all studied compounds was observed, as shown in Figure 2. The maximum solubility values achieved in aqueous media depended on the compound’s structural characteristics and its compatibility with the specific surfactant, thereby enhancing its apparent solubility in water.

**Figure 2:**
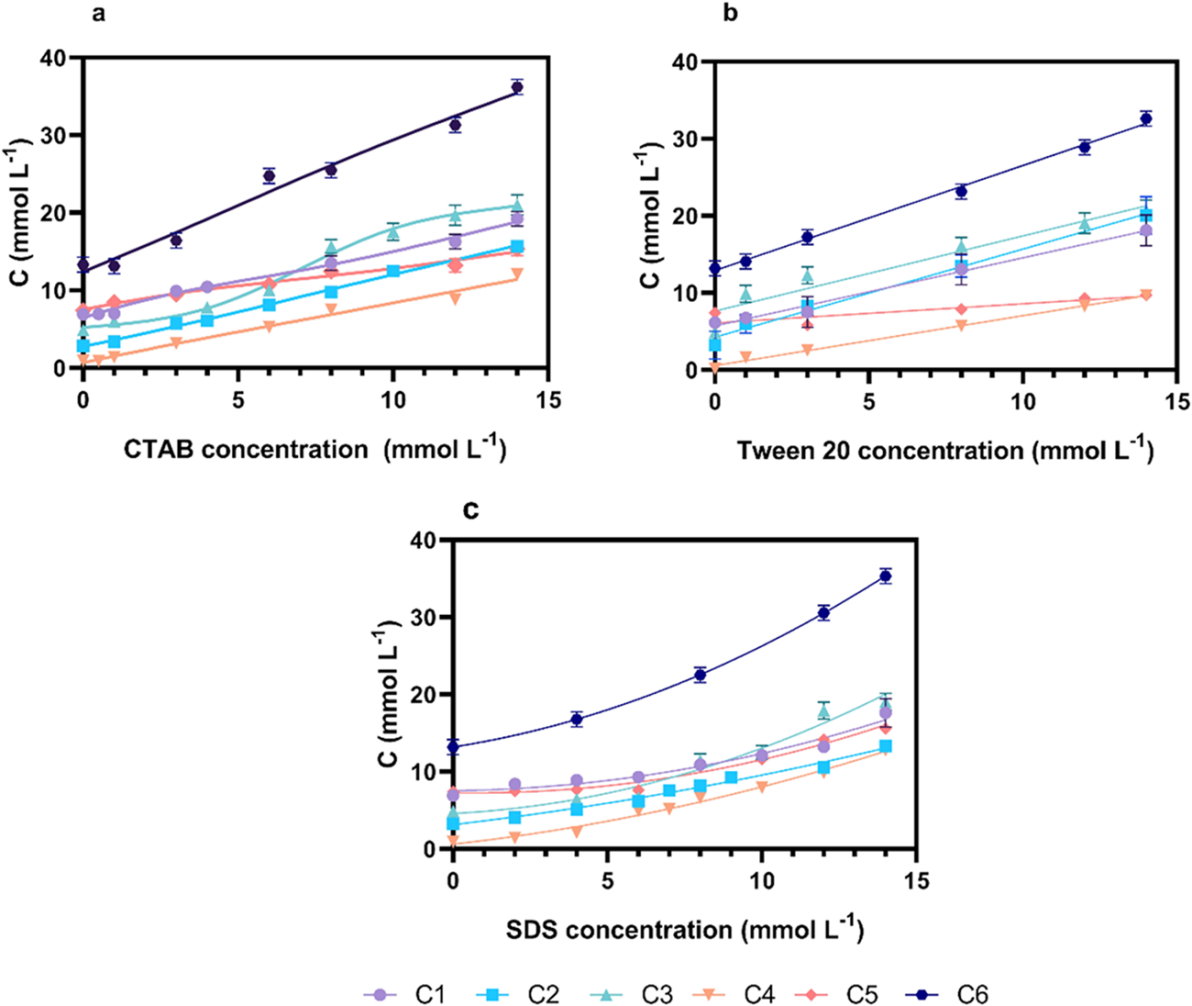
Solubility in water of the six phosphorus–selenide compounds with surfactants: (a) CTAB, (b) Tween 20, and (c) SDS, in the range of 0–14 mmol L^–1^ at 25 °C. The lines connecting the data points are merely guides to the eye and do not result from any fitting procedure. For interpretation of the references to color in this figure legend, the reader is referred to the web version of this article.

In general, the solubility of the compounds in water increased linearly with surfactant concentration, as observed for CTAB (Figure 2a) and Tween 20 (Figure 2b). In contrast, SDS (Figure 2c) demonstrated a nonlinear behavior. An initial linear segment is observed, followed by a change in slope corresponding to a second linear region. This slope aligns with the critical micelle concentration (CMC) of pure SDS, reported as 8.2 mM at 25 °C.^44^ A more specific and comprehensive analysis of the CMC and critical aggregation concentration (CAC) for these mixtures will be presented in Section 3.4.

For the Tween 20 and the CTAB, the CMC is reached in low surfactant concentration (0.89 mmol L^–1^ and 0.00195 mmol L^–1^ at 25 °C, respectively, for surfactant pure),^34,47^ which explains a linear solubility behavior with these surfactants. In this case, micelle formation occurs almost immediately in the studied range, so that solubilization is dominated by micellar incorporation of the compounds. The structural characteristics of Tween 20 (nonionic, polyoxyethylene headgroup) and CTAB (cationic, C16 chain) favor the formation of stable micelles, minimizing any premicellar regime and resulting in a predominantly linear increase in solubility with surfactant concentration.

The solubility of each compound (Figure 2) in water starts from the surfactant-free concentration point, i.e., the pure compound in water. The compound C6 is the most soluble (13.20 ± 0.98 mmol L^–1^), which is related to its chemical structure since, unlike the other compounds, it does not have associated aromatic groups. Likewise, when surfactant is added, its solubility increases. When the surfactant concentration increases until 14 mmol L^–1^, it is observed to almost triple the compound concentration in water, regardless of the surfactant′s nature. C4 has a lower solubility (0.93 ± 0.21 mmol L^–1^) among the compounds studied. When the surfactants were added, the solubility of C4 in water increased by approximately 14 times.

Organophosphorus compounds with diethylphosphonate groups generally exhibit moderate to good solubility in aqueous media due to the polarity of the diethylphosphonate group. However, their solubility can be significantly reduced when bulky hydrophobic chains are present, as these groups hinder their interaction with the polar solvent.

The solubility differences with the same surfactant were analyzed at 14 mmol L^–1^. For the cationic surfactant CTAB, compound C6 showed the highest solubility, while C1 and C3 exhibited no significant difference between them, similar to C5 and C2; thus, the solubility order was C6 > C1, C3 > C2, C5 > C4. The C6 compound, being aliphatic, exhibits lower polarity compared to aromatic compounds due to the absence of an aromatic ring. This property makes them more compatible with the hydrophobic environment of the colloidal structure formed with CTAB. C1 contains a *p-*methoxy group that increases the molecule’s hydrophobicity, affecting its compatibility with the hydrophobic tails of the surfactant and forming a self-assembly system. As a result, it did not show a significant difference compared to C3, which lacks substituents on the aromatic ring. In contrast, C2, with a *p*-methyl group, exerts a weaker influence than the *p-*methoxy group. Compounds with bulkier (C5 and C4) substituents exhibit steric hindrance, negatively affecting their interaction and solubility with CTAB.

For the SDS at 14 mmol L^–1^, C6 was the most soluble compound and showed significant differences compared to all other compounds (*p* < 0.05). C1, C3, and C5 compounds showed no significant difference (*p* > 0.05) between them, and those compounds presented greater solubility than C4 and C2, which did not present a difference between themselves. The order of solubility in SDS remains with the compounds in C6 > C1, C3, C5 > C2, C4. C6 exhibited higher solubility due to its efficient interaction with the surfactant tails, allowing it to remain encapsulated within the colloidal structure. On the other hand, a significant difference was observed with aromatic compounds, which, when compared to C6, were less available in water. Due to their planar and rigid structure, they have less flexibility than aliphatic compounds when interacting with the surfactant tails. C1 contains a *p-*methoxy group which increases the hydrophobicity and compatibility within the colloidal structure formed, similar to C3. In contrast to CTAB, C5 showed greater compatibility with SDS, enhancing its solubility despite its larger size. This suggests that the influence of its methoxy groups within the molecule and its compatibility with water and SDS played a more significant role.

For the neutral surfactant Tween 20, the solubility in water order was C6 > C1, C2, C3 > C4, C5, with C6 maintaining the highest solubility and C1, C2, and C3 showing no significant difference between each other, while C5 and C4 presented the lowest solubility in water. In this case, the effect of para-position donor groups (methyl and methoxy) did not significantly differ between them or with the unsubstituted aromatic ring. Meanwhile, C4 and C5 demonstrated that their molecular size likely influenced their compatibility with Tween 20 micelles, possibly due to steric repulsion effects.

As a result, ionic surfactants (CTAB and SDS) proved to be the most efficient dispersants in all cases. Their electrostatic interactions with the compounds were favorable, preventing destabilization of the surfactant polar heads within the colloidal structure. This stability facilitated effective dispersion of the compounds with the surfactant, enabling the formation of colloidal structures.

### 3.2 Size and Colloidal Stability

The hydrodynamic sizes of all pure phosphorus–selenium compounds were measured before to surfactant addition. Compounds C1, C2, C3, C4, and C6 exhibited self-aggregation, which formed aggregates with broad dispersions (Figure 3), whereas compound C5 could not form a stable self-assembled structure due to its chemical and electrostatic characteristics.

**Figure 3:**
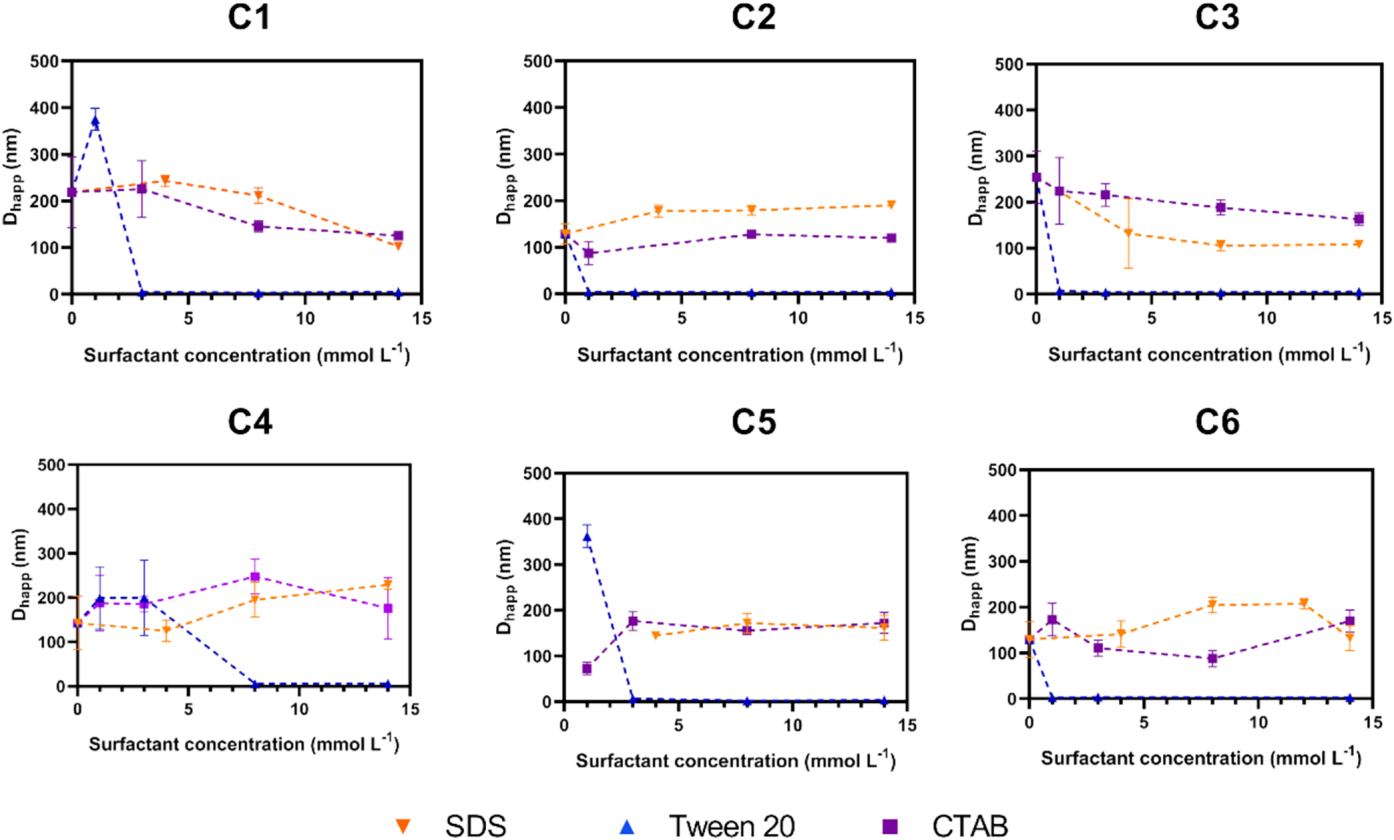
Hydrodynamic diameter (by number) of the six phosphorus–selenium compounds in CTAB (purple), SDS (orange), and Tween 20 (blue), within the concentration range 0–14 mmol L^–1^, at 25 °C. For interpretation of the color references in this figure legend, the reader is referred to the web version of this article.

For CTAB and SDS formulations, particle sizes ranged from 100 to 250 nm, depending on the compound and surfactant concentration. This is consistent with the formation of nanoemulsions that stabilize and solubilize the system through colloidal structures, where the liquid compound behaves as the oil phase, whose stabilization is spontaneously favored by the presence of surfactants in the aqueous phase. These sizes are larger than typical micelle diameters (∼4–5 nm for SDS and 3–6 nm for CTAB),^35,47^ indicating that the surfactants can form nanoemulsions depending on the interacting molecules.^35,48,49^ The polydispersity index (PDI) values ranged from 0.12 to 0.300, indicating a relatively narrow size distribution and satisfactory colloidal stability^50^

Particularly at concentrations above 5 mmol L^–1^, Tween 20 significantly reduced the hydrodynamic diameter for all compounds, with values comparable to those reported for Tween 20 alone (2.87 ± 0.05 nm at 0.1–10 mmol L^–1^).^48^ However, limited solubility improvement was observed for C4 and C5 dispersed in Tween 20, as indicated by larger particle sizes and broader distributions at low concentrations of Tween 20. These observations suggest that micelle formation may occur, but stabilization is not sustained, as confirmed by ζ-potential results (Figure 4a) and the phase separation after a few days.

**Figure 4:**
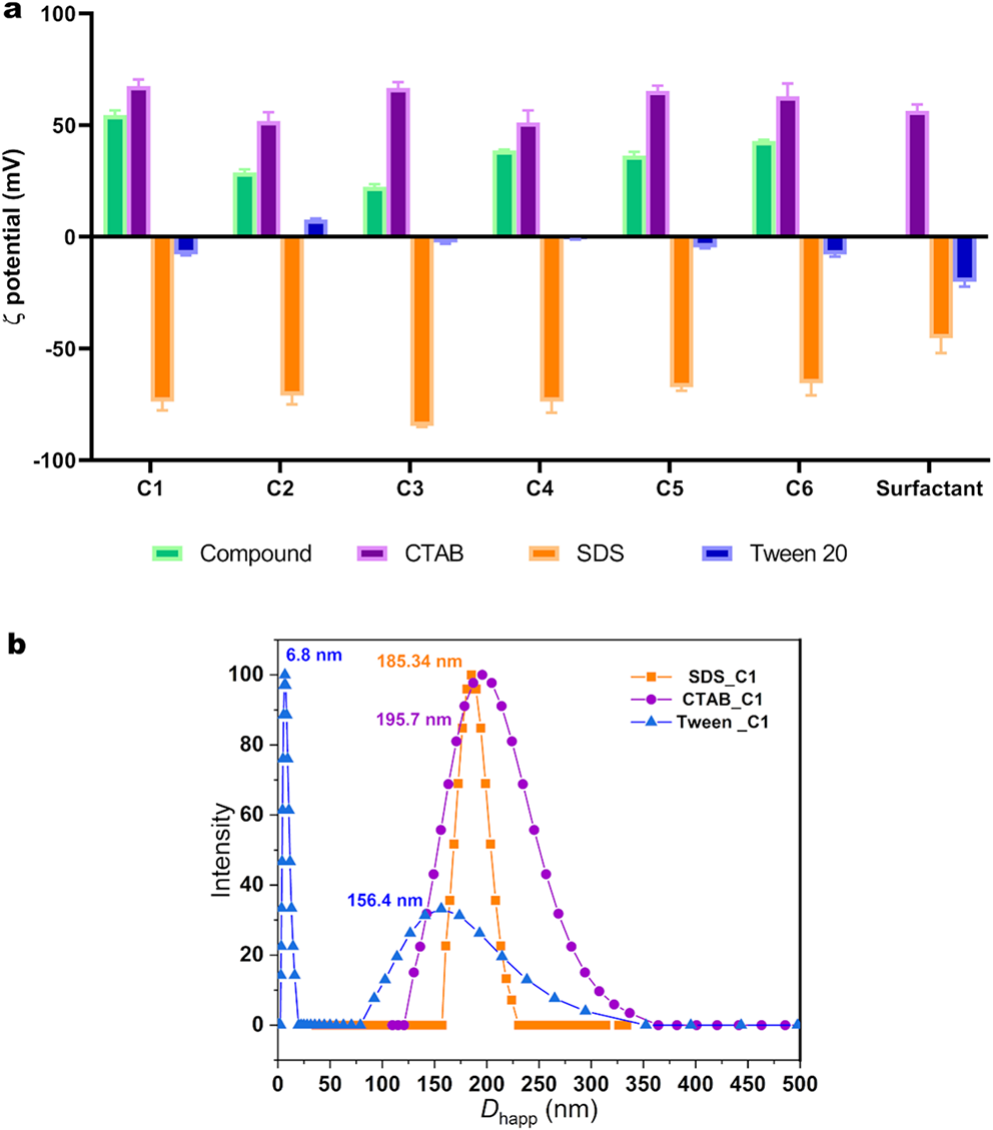
(a) ζ-Potential (ζ) measurements of the six phosphorus–selenium compounds in CTAB (purple), SDS (orange), and Tween 20 (blue) formulations (14 mmol L^–1^) at 25 °C (Values of ζ > 50 mV are considered apparent ζ-potentials). (b) Hydrodynamic diameter (by intensity) of compound C1 in CTAB (purple), SDS (orange), and Tween 20 (blue) at 14 mmol L^–1^ and 25 °C. For interpretation of the references to color in this figure legend, the reader is referred to the web version of this article.

To further understand the behavior of the Tween-based system, DLS measurements by intensity were examined. Unlike CTAB and SDS systems, which displayed a single population across surfactant concentrations, Tween systems exhibited two distinct populations (Figure 4b), indicating progressive aggregation and phase separation. Low micellar incorporation was observed, with micelles retaining sizes similar to pure Tween 20. In the aqueous medium, these compounds primarily act as a depletion agent and solvation competitor, reducing the hydration and thickness of the ethylene oxide layer responsible for steric stabilization in Tween 20. Consequently, in the absence of surface charge (ζ ≈ 0) and without sufficient steric protection, micellar collisions lead to slow flocculation and subsequent phase separation. Additionally, the self-association of the aromatic moieties of the molecule may accelerate instability, promoting localized precipitation.^51,52^ The potential stabilization of Tween 20 systems could be enhanced by adjusting the pH or blending with other surfactants such as Tween 80, Span 20, or cholesterol.^51–55^

The positive ζ-potentials of the phosphorus–selenium compounds can be explained by the presence of phosphate groups with ether substituents, whose oxygen atoms may undergo protonation or preferential cation binding. In addition, the aromatic rings favor hydrophobic aggregation, which can shield deprotonated groups and expose ether-rich domains at the interface. These combined effects result in an apparent positive surface potential.

In absolute value, the ζ-potential for the compounds in CTAB and SDS increased once the compound interacted with the surfactant, forming a nanoemulsion. ζ-Potentials above ± 30 mV indicate colloidal stability,^56,57^ which was observed for phosphorus selenide compounds in CTAB and SDS. Therefore, stable dispersions were obtained using the phosphorus selenide compounds and the ionic surfactants. The mean ζ-potential observed for all compounds with SDS and CTAB is noteworthy, suggesting preferential interaction with the hydrophobic tails rather than the ionic head groups.

### 3.3 Atomic Force Microscopy

The morphology and size distribution of the nanoemulsion formed by the phosphorus–selenium compound C3 in SDS 14 mmol L^–1^ were analyzed using atomic force microscopy (AFM). The AFM images (Figure 5a,5b) reveal spherical particles homogeneously distributed across the substrate, in a short and large scanning area. The observed particle diameters are consistent with the hydrodynamic sizes measured by dynamic light scattering, ranging approximately from 100 to 250 nm (Inset Figure 5a), which supports the formation of a stable nanoemulsion.^58,59^ These observations align with the colloidal stability inferred from the PDI index values and the favorable ζ-potential measurements.

**Figure 5:**
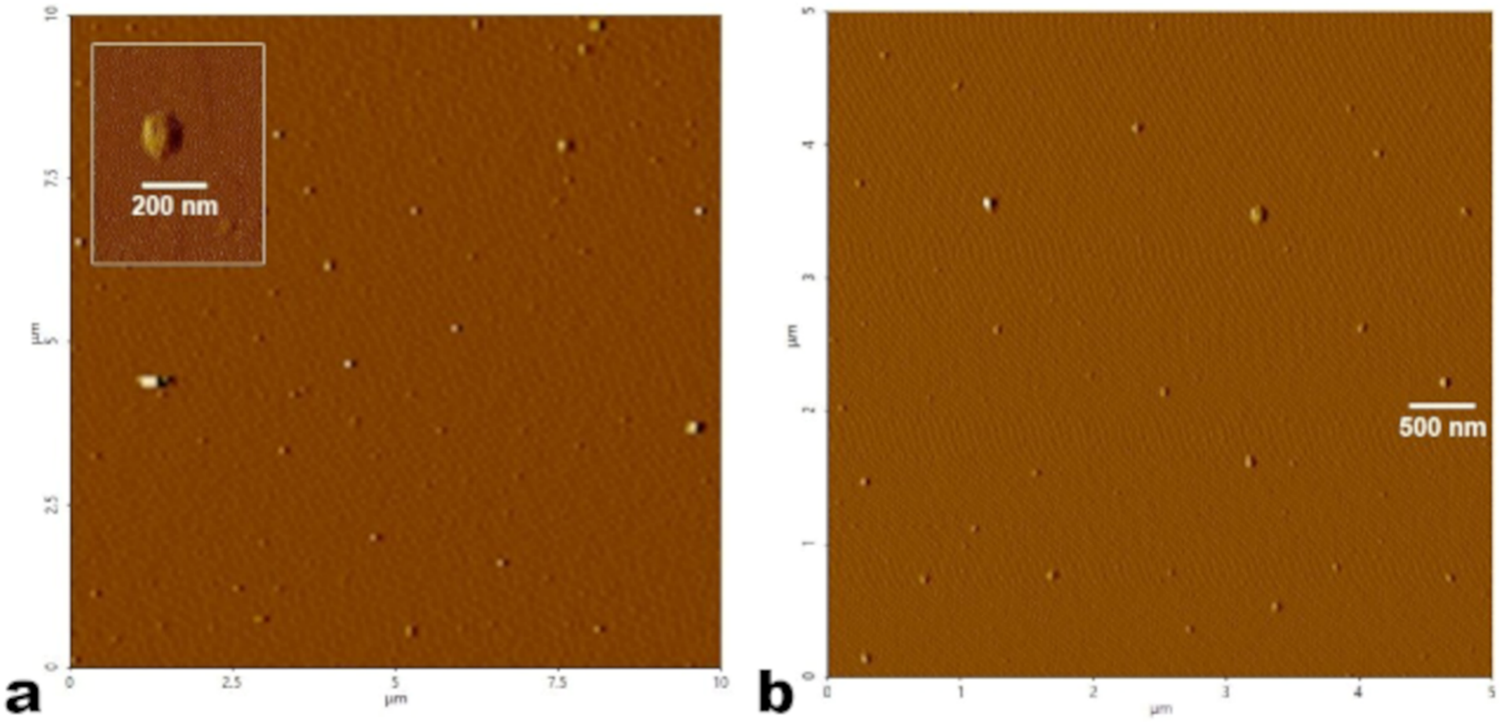
Topographic micrographs obtained by AFM for the C3_SDS mixture on the mica substrate. (a) Scale (10 μm × 10 μm, dynamic mode) and (b) magnification view (5 μm × 5 μm, dynamic mode).

The AFM data further support that the phosphorus selenide compound interacts with SDS to form nanoemulsions rather than simple micelles, thereby providing stabilization and improved dispersion in aqueous media. The spherical morphology and uniform distribution suggest that the surfactant effectively reduces interfacial tension and prevents particle coalescence. This characteristic is crucial for potential applications in biological or agrochemical formulations.

### 3.4 CAC Determination and Thermodynamic Parameters

Conductivity measurements are highly effective in studying association behavior and detecting structural changes in ionic systems. Figure 6 presents representative plots of specific conductivity (κ) as a function of CTAB or SDS surfactant concentration (mmol L^–1^) at 25 °C. The purple curves represent the measurements for each pure surfactant in water, while, the blue and green curves are the measurements of the ionic surfactants with the compounds at two fixed concentrations (3 mmol L^–1^ and 10 mmol L^–1^). Plots exhibit two linear regions with distinct slopes, pre and post micellar regions, where the breakpoint corresponds to the CMC for the pure surfactant.^33,46^

**Figure 6:**
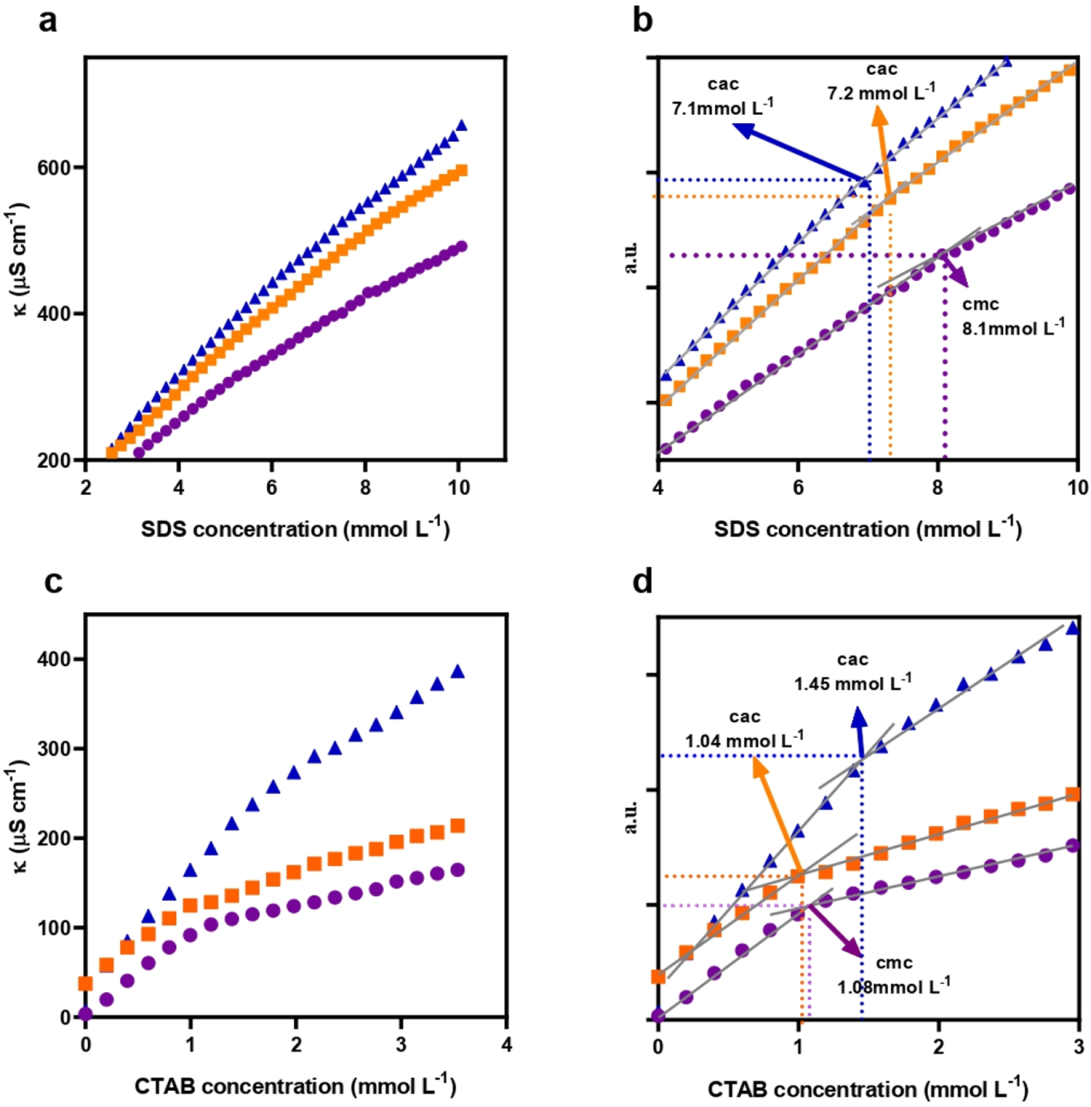
Conductivity measurements for C2 compounds with three surfactant concentrations: 0 mmol L^–1^ (purple), 3 mmol L^–1^ (orange), and 10 mmol L^–1^ (blue) at 25 °C. Panels: (a) C2 with SDS; (b) zoom of C2 with SDS showing gray linear fit; (c) C2 with CTAB; and (d) zoom of C2 with CTAB showing gray linear fit. For interpretation of the colors in this figure, the reader is referred to the web version of this article.

By comparison, the slope change is less pronounced for the mixtures, suggesting a more gradual transition between the pre- and postaggregation regions. This behavior is consistent with the formation of nanoemulsions rather than conventional micelles, resulting in a broader distribution of aggregate sizes and a reduced conductivity change at the aggregation threshold. Additional curves are provided in the Supporting Information (Figure S1).

The CMC values obtained for SDS and CTAB are consistent with the literature data (Table S1). For SDS, the CMC is typically reported at approximately 8.1 mmol L^–1^ at 25 °C, whereas for CTAB it is found around 1.0 mmol L^–1^ at 25 °C.^45,60,61^ For the mixtures, apparent CAC values and the aggregation Gibbs free energy were calculated using eq 1, even though nanoemulsions rather than micelles were formed and the conductivity curves did not exhibit a clear inflection point. These approximate calculations are presented in Table S1, together with an explanation provided for comparative purposes of these systems.

### 3.5 Characterization with FTIR and NMR

FTIR analysis was performed to characterize the colloidal systems, specifically to verify any possible reactions or modifications with the surfactant that could lead to degradation or alteration of the original compound, thereby preventing its use. As observed in Figure 7 for C1, the band at 3580–3200 cm^–1^ is related to the stretching of the OH– group, mainly due to water traces. In the 2930–2850 cm^–1^ range, a band related to axial deformations of CH links from −CH_3_ and −CH_2_ groups indicated the presence of surfactant. This band is not as strong for the compound alone, which does not contain alkyl chains as long as surfactants have, but it can be observed that it has the C–H band of aromatics (3000 cm^–1^). This result was expected because this band is characteristic of the Tween 20, CTAB, and SDS hydrophobic tails, which were also observed in the mixtures (Figure 7b–g).

**Figure 7:**
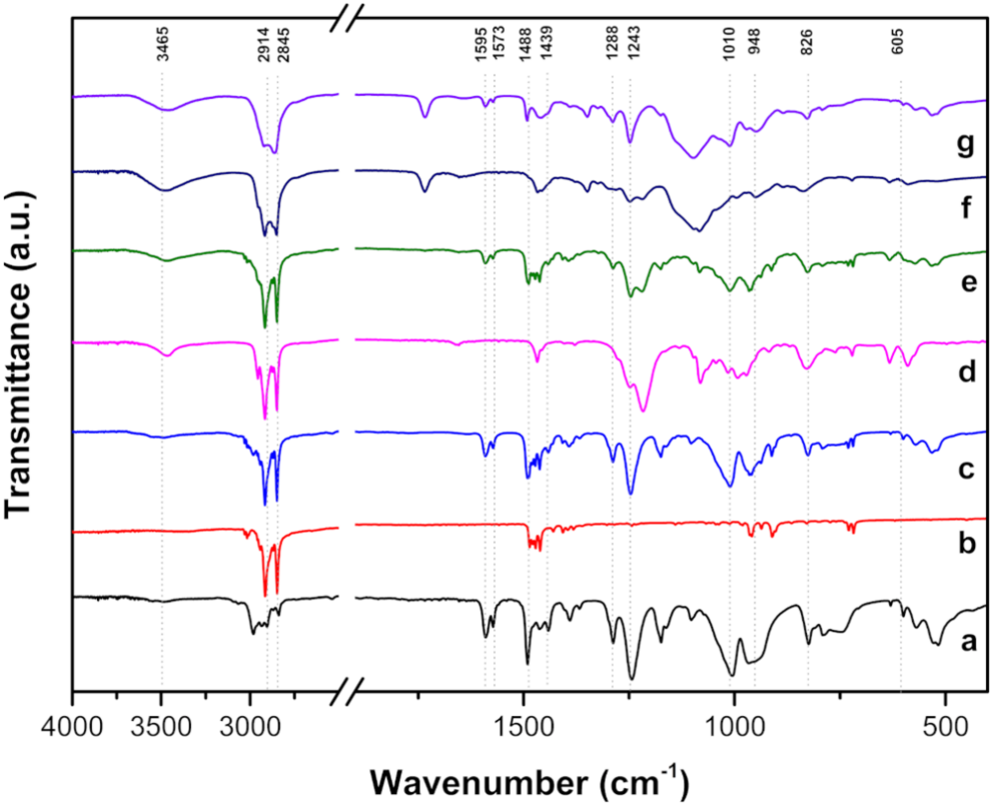
FTIR analysis for the phosphorus selenide compounds: (a) diethyl Se-(4-methoxyphenyl) phosphorus selenide (C1), (b) CTAB, (c) C1 and CTAB, (d) SDS, (e) C1 and SDS, (f) Tween 20, and (g) C1 and Tween 20. For interpretation of the references to color in this figure legend, the reader is referred to the web version of this article.

The band at 1595–1575 cm^–1^ present in entries a, c, e, and g of Figure 6 corresponds to the stretching of the C=C group in aromatic rings found in C1. In these same samples, other peaks related to the pure compound were identified, such as those at 1420–1490 cm^–1^, corresponding to the −CH_2_– group; 1200–1300 cm^–1^ related to the P=O bond; and phosphate, covalent, aromatic, and aliphatic ester groups are observed in the region of 948–1010 cm^–1^. The 800 – 850 cm^–1^ region relates to *p*-aromatic substitution. The antisymmetric C–Se stretching at 605 cm^–1^ ^62^ was also found in these entries. FTIR spectra were also recorded for the other compounds studied (C2 to C6), showing characteristic peaks for each compound, as shown for C1 in Figure 7 (Figures S2–S6).

We also performed ^1^H NMR experiments to evaluate the possible reactions of the phosphorus selenide compounds with surfactants. The ^1^H NMR spectrum of diethyl Se-phenyl phosphorus selenide (C3) showed the presence of characteristic signals for the alkyl hydrogen of the ethyl group at 1.25 ppm (CH_3_) and 4.20 ppm (CH_2_), presented in Figure 8. In addition, the signals of the hydrogen of the aromatic system directly bonded to the selenium atom are observed as a multiplet between 7.25 and 7.70 ppm (Figure 8a). Figure 8b shows the ^1^H NMR of cetyltrimethylammonium bromide (CTAB). In this spectrum, the signal at 0.9 ppm corresponds to the methyl group of the alkyl chain, the singlet at 1.3 ppm refers to the 14 CH_2_ of the alkyl chain, a singlet at 3.4 ppm refers to the three methyl groups directly bonded to the nitrogen atom and at 3.6 ppm a signal referring to the CH_2_ of the alkyl chain directly bonded to the nitrogen atom. We also performed an NMR experiment on a sample obtained by performing CTAB solubilization experiments with Se-phenyl phosphorus selenide (C3). In this experiment, we observed that all the characteristic signals of both the selenium compound and CTAB were present in the spectra without any displacement of the signals or any change in the multiplicity of the signals (Figure 8c). The ^1^H NMR experiments demonstrate that the selenium compound and CTAB coexist in the system without undergoing any chemical reaction, as evidenced by the unchanged position and multiplicity of their characteristic spectral signals. (Additional FTIR and ^1^H NMR are provided in the Supporting Information).

**Figure 8:**
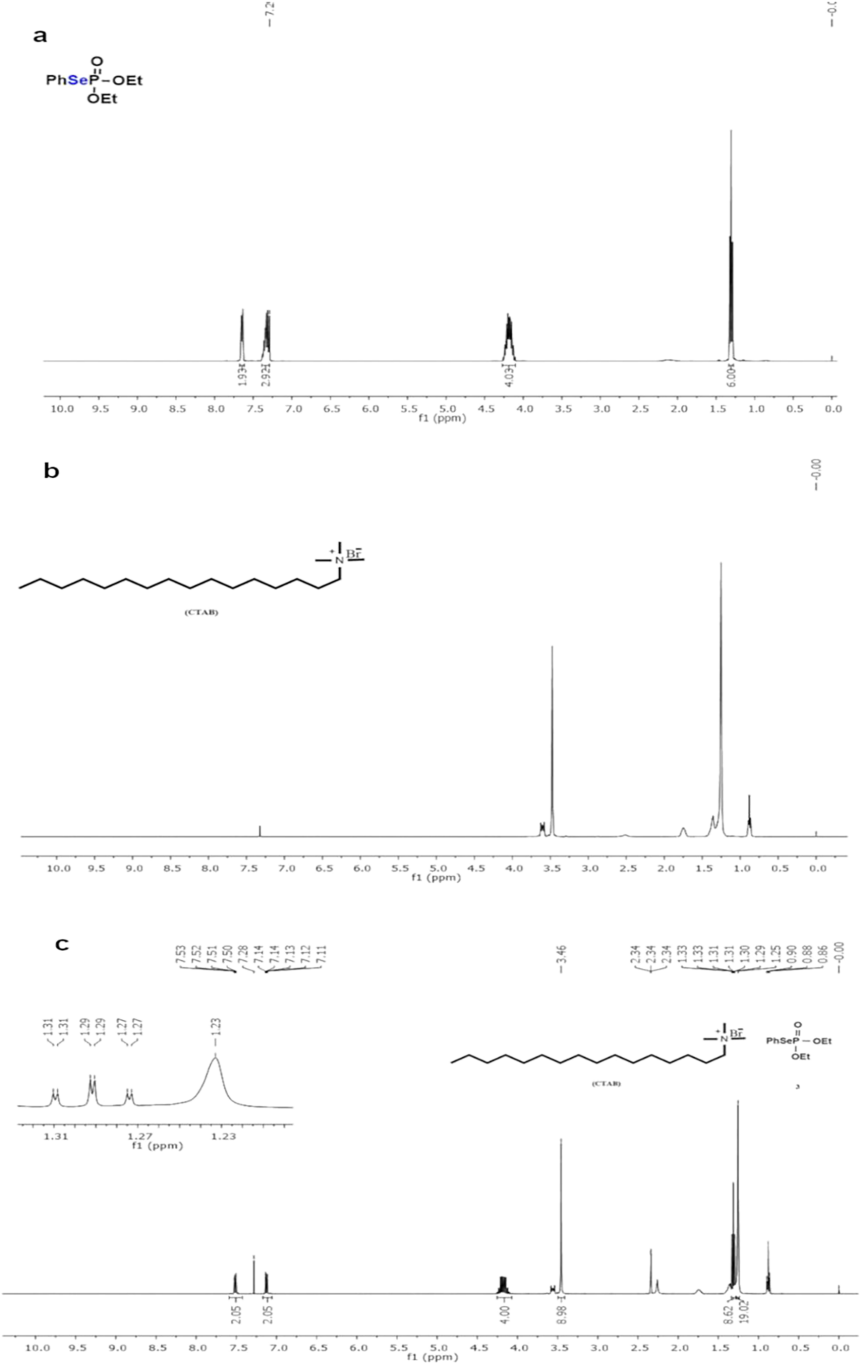
^1^H NMR spectra for: (a) the Se-phenyl phosphorus selenide (C3); (b) CTAB; (c) the Se-phenyl phosphorus selenide (C3) with CTAB.

## 4 Conclusions

This study investigated the interactions between different surfactants and phosphorus selenide compounds, demonstrating that all tested surfactants enhanced the compounds’ solubility in water. Depending on the surfactant, colloidal structures with distinct hydrodynamic sizes were formed. Specifically, Tween 20 resulted in particle sizes smaller than 5 nm, while SDS and CTAB produced colloidal structures with an average size of approximately 200 nm and significantly high ζ-potential values, indicating the formation of stable spontaneous nanoemulsions.

The extent of solubility enhancement varied according to the molecular characteristics of the compounds, with a general increase observed upon reaching the surfactant’s critical aggregation concentration (CAC). Among the studied compounds, the aliphatic compound C6 exhibited the greatest solubility enhancement, whereas the aromatic compound C4, containing an m-trifluoromethyl group, showed the lowest. ζ-Potential, DLS, and AFM measurements confirmed the formation of spontaneous nanoemulsions with SDS and CTAB, which displayed higher stability compared to the smaller aggregates formed with Tween 20. In contrast, conductivity measurements did not exhibit the expected inflection point required for rigorous CAC or β determination, a deviation consistent with the behavior typically observed in nanoemulsion.

FTIR and NMR analyses confirmed the absence of chemical reactions between the surfactants and the compounds, demonstrating the effective incorporation of the compounds into the nanoemulsions while preserving their original chemical structure. These findings contribute to the development of novel formulations for the application of phosphorus selenide compounds in aqueous media, ensuring improved solubility and stability. Furthermore, the use of widely available and cost-effective surfactants enhances the feasibility of these formulations for practical applications.

## Supplementary Material



## Notes

NMR and FTIR spectra; conductivity measurements; table of critical micelle concentration (CMC), critical aggregation concentration (CAC), and standard Gibbs free energy of aggregation (Δ*G*°_agg_) at 25 °C (PDF)
