# Supplementary material for: Novel Spontaneous Nanoemulsions for Phosphorus Selenide Compounds: Toward Enhanced Solubility and Stable Aqueous Formulations
Source: ACS Omega. 2025 Nov 11;10(46):56566–77. doi: 10.1021/acsomega.5c08867 (PMC12658836; doi:10.1021/acsomega.5c08867)
Supplement: Supplementary file 1 [file ao5c08867_si_001.pdf]

NOVEL SPONTANEOUS NANOEMULSIONS FOR PHOSPHORUS SELENIDE COMPOUNDS: TOWARD ENHANCED SOLUBILITY AND STABLE AQUEOUS FORMULATIONS

Romelly Eugenia Rojas Ramírez[a,*], Daiani Canabarro Leite[b,*], Ana Maria Recchi[a], Nadia Carollayn Correa da Silva, Yago Cezar Bastianello, Fabíola Caldeira dos Santos[a], Tielle Moraes de Almeida[b], Gilson Zeni[a, *]

[a] Department of Biochemistry and Molecular Biology, UFSM, Brazil

[b]Department of Physics. UFSM, Brazil

[*]Corresponding author. E-mail addresses (romelly@gmail.com) (R.E.R. Ramirez), gzeni@ufsm.br (G.Zeni), and (daiani.leite@ufsm.br) (D. C. Leite)

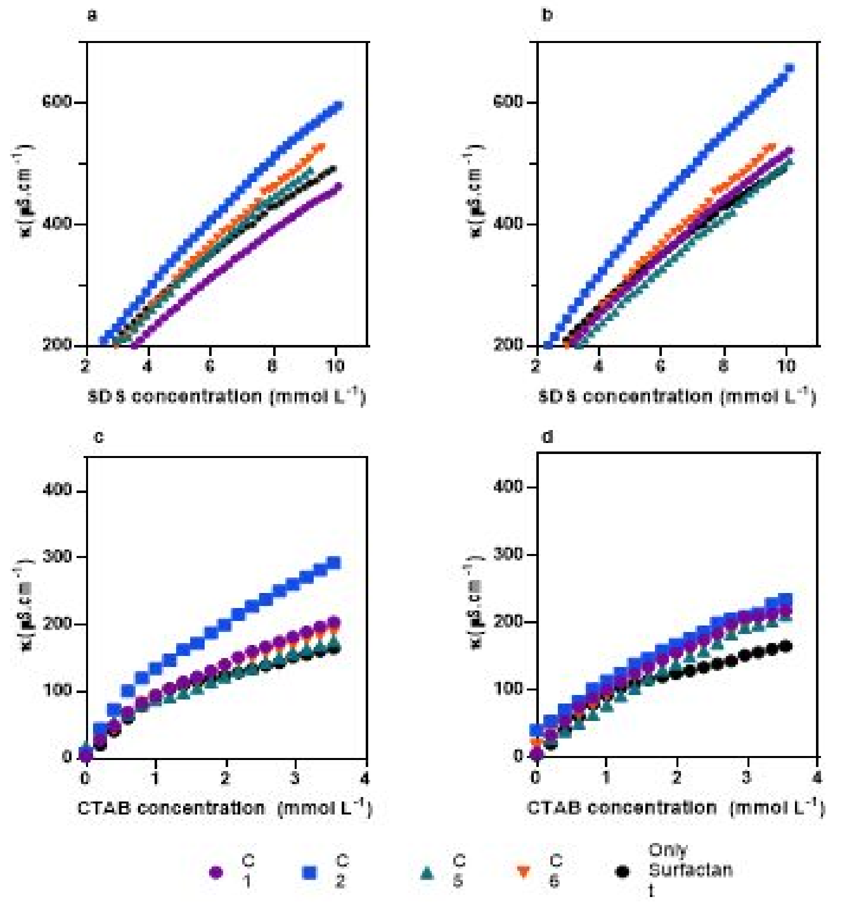

Figure S1. Conductivity measurements for C1, C2, C5 and C6 for two concentrations at 25°C with CTAB and SDS surfactants: (a) and (b) 3 and 10 mmol L^{-1} of compound in SDS; (c) and (d) 3 and 10 mmol L^{-1} of compound in CTAB. For interpretation of the references to color in this figure legend, the reader is referred to the web version of this article.

Table S1. CMC, CAC, and aggregation Gibbs free energy ($\Delta G°_{agg}$) at 25 °C, measured by conductivity and calculated with equation (1).

Sample	*Se_Compounds (mmol L⁻¹)*	*SDS*			*CTAB*		
		**CMC/ CAC (mmol L^{-1})**	**β**	**ΔG°agg (kJ mol^{-1})**	**CMC/ CAC (mmol L^{-1})**	**β**	**ΔG°agg (kJ mol^{-1})**
**SDS**	**0**	8.1±0.6	0.500±0.017	-31.3±0.7	-	-	-
**CTAB**	**0**	-	-	-	1.08±0.06	0.264±0.009	-46.6±1.6
**C1**	**3**	7.4±1.2	0.776±0.011	-27.0±0.6	0.77±0.11	0.420±0.045	-43.4±2.1
	**10**	7.2±0.7	0.747±0.008	-27.8±0.4	0.77±0.15	0.521±0.050	-41.0±3.2
**C2**	**3**	7.2±0.8	0.662±0.011	-29.3±0.6	1.04±0.09	0.434±0.037	-42.5±3.6
	**10**	7.1±0.7	0.758±0.010	-27.5±0.5	1.45±0.22	0.717±0.035	-33.5±1.7
**C3**	**3**	6.0±0.8	0.808±0.017	-26.9±0.7	0.96±0.12	0.427±0.021	-42.7±2.1
	**10**	5.3±0.6	0.791±0.024	-27.7±0.9	0.77±0.05	0.527±0.010	-40.8±0.8
**C4**	**3**	7.0±0.5	0.720±0.008	-28.4±0.4	0.73±0.10	0.474±0.026	-35.7±2.1
	**10**	5.6±1.0	0.822±0.013	-26.8±0.7	0.93±0.11	0.379±0.020	-44.2±2.4
**C5**	**3**	7.0±0.6	0.673±0.011	-29.5±0.5	0.72±0.26	0.633±0.040	-38.1±2.7
	**10**	7.6±1.0	0.898±0.007	-24.2±0.4	0.85±0.19	0.639±0.028	-37.4±1.8
**C6**	**3**	5.1±0.4	0.865±0.022	-26.1±0.7	0.79±0.09	0.452±0.018	-42.8±1.8
	**10**	5.2±0.5	0.779±0.026	-27.9±1.0	1.27±0.24	0.628±0.035	-36.2±2.1

The critical aggregation concentration (CAC) and the approximate value of the standard free energy of aggregation (ΔG°agg) for the mixture were calculated using equation (1), and the results are reported in Table S1. The obtained free energy values indicate that the process is favorable for a spontaneous system.

With the addition of the phosphorus selenide compounds, a decrease in the CAC value was observed for both the cationic and anionic surfactants. This decrease in CAC means a favorable interaction between compounds and the surfactant. However, it was observed that for CTAB associated with compound C2, at 10 mmol L^{-1}, a significantly higher CAC value was found ($p < 0.05$), which could imply a possible influence of these compounds on the formation of the micelles and aggregates, increasing the concentration at which the CTAB micelle is formed. C2 is a compound with a *p*-methyl group in the aromatic ring, which increases electron density and influences interactions with the cationic heads of

CTAB, thereby increasing repulsive interactions that may be intervene in the aggregation process. For C6 with CTAB, the CAC value was also higher for the 10 mmol L^{-1} concentration. C6 is a compound without aromatic rings and with the lowest molar mass. Therefore, the number of molecules at this concentration is higher than that of the other systems. This may be the reason for its influence on the colloidal aggregation, as the increased number of molecules in this concentration enhances the likelihood of interactions with the surfactant head groups. The SDS's most significant influence on the CAC was with C6 ($p < 0.05$), resulting in an average value of 5.59 ± 0.06 mmol L^{-1} for both concentrations.

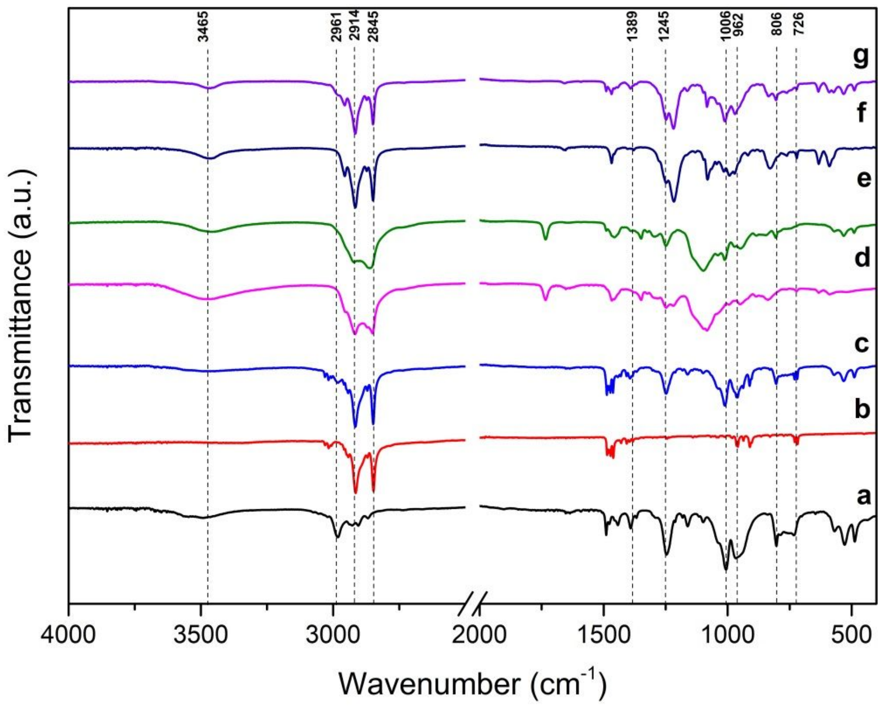

Figure S2. FTIR analysis for the phosphorus selenide compounds: a) C2 diethyl Se-*p*-tolyl phosphorus selenide, b) CTAB, c) C2 and CTAB, d) Tween 20, e) C2 and Tween, f) SDS and g) C2 and SDS. For interpretation of the references to color in this figure legend, the reader is referred to the web version of this article.

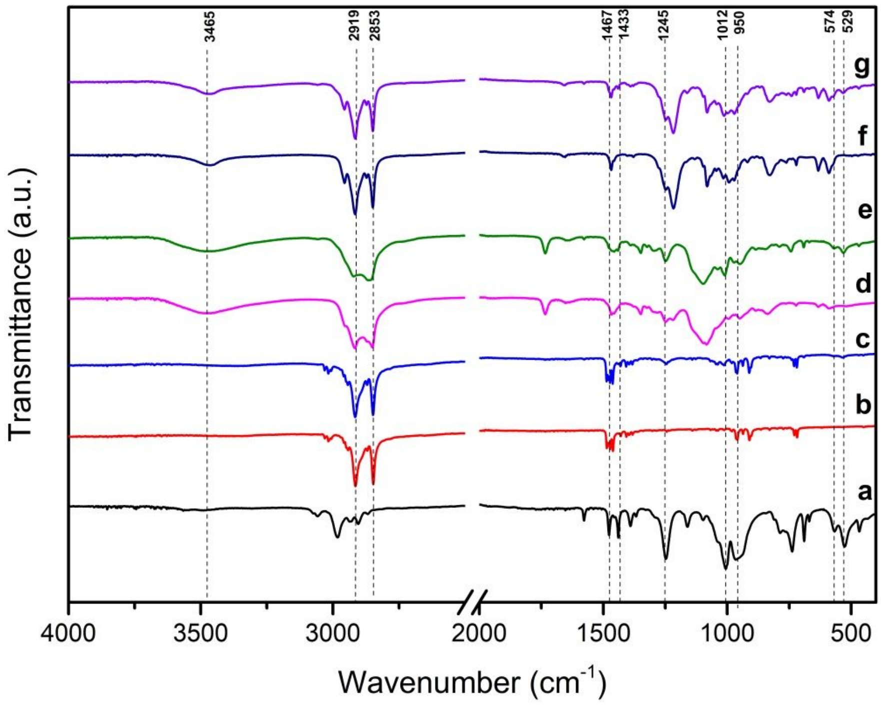

Figure S3. FTIR analysis for the phosphorus selenide compounds: a) C3 diethyl Se-phenyl phosphorus selenide, b) CTAB, c) C3 and CTAB, d) Tween 20, e) C3 and Tween, f) SDS and g) C3 and SDS. For interpretation of the references to color in this figure legend, the reader is referred to the web version of this article.

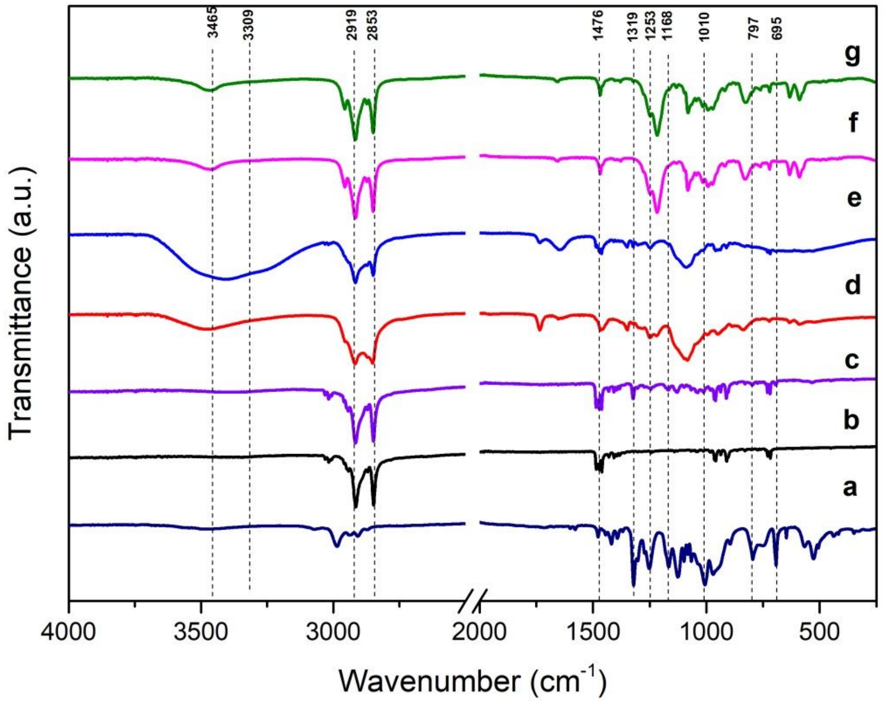

Figure S4. FTIR analysis for the phosphorus selenide compounds: a) C4, diethyl Se-(3-(trifluoromethyl)phenyl) phosphorus selenide, b) CTAB, c) C4 and CTAB, d) Tween 20, e) C4 and Tween, f) SDS and g) C4 and SDS. For interpretation of the references to color in this figure legend, the reader is referred to the web version of this article.

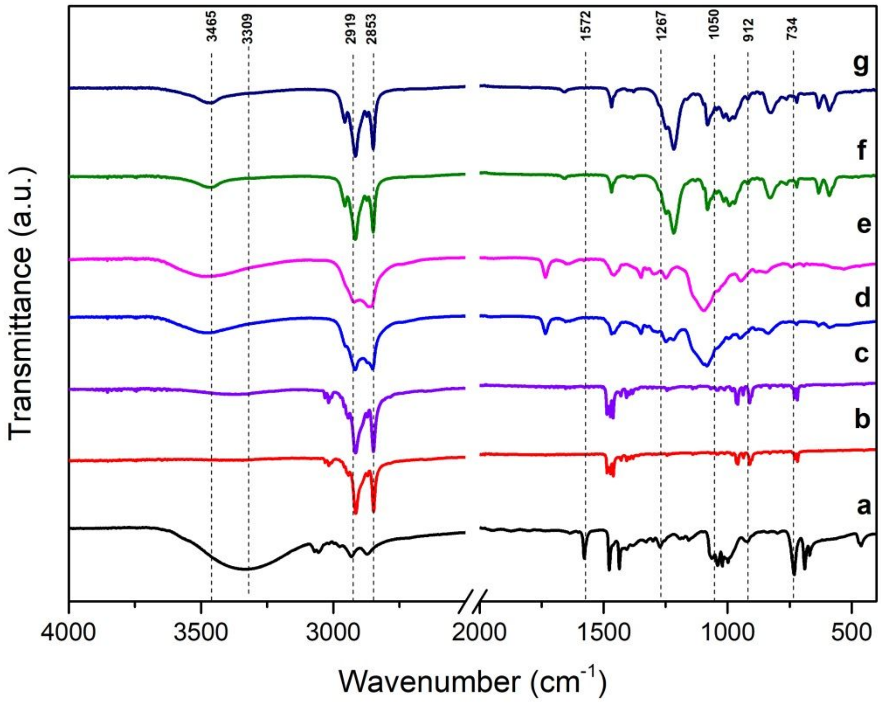

Figure S5. FTIR analysis for the phosphorus selenide compounds: a) C5, tris(2-(phenylselanyl)ethyl) phosphate, b) CTAB, c) C5 and CTAB, d) Tween 20, e) C5 and Tween, f) SDS and g) C5 and SDS. For interpretation of the references to color in this figure legend, the reader is referred to the web version of this article.

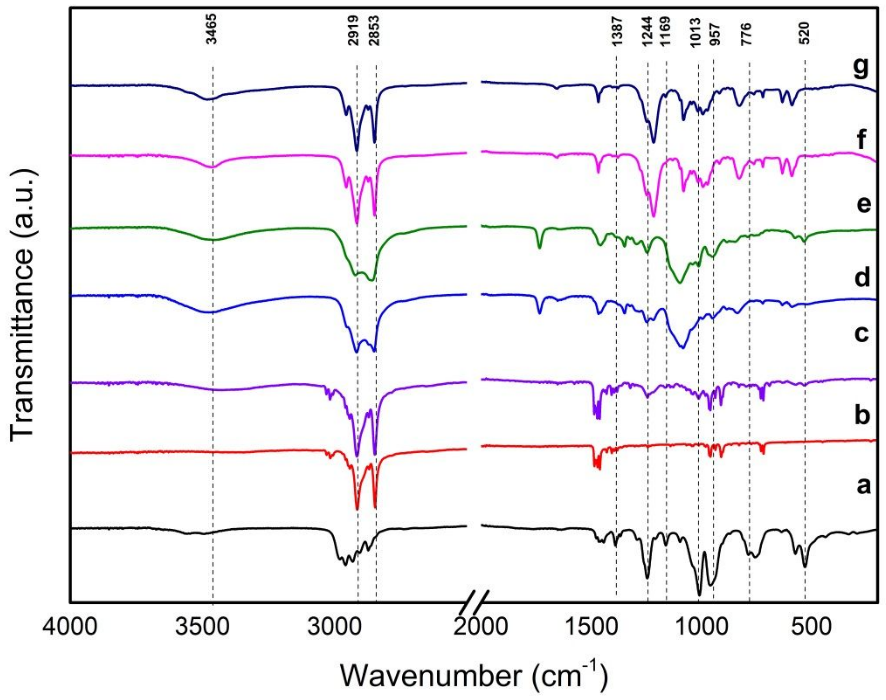

Figure S6. FTIR analysis for the phosphorus selenide compounds: a) C6, Se-butyl-diethyl phosphorus selenide b) CTAB, c) C6 and CTAB, d) Tween 20, e) C6 and Tween, f) SDS and g) C6 and SDS. For interpretation of the references to color in this figure legend, the reader is referred to the web version of this article.

Representative Examples of Nuclear Magnetic Resonance Analyses

The ^{1}HNMR (600 MHz) and ^{13}CNMR (150 MHz) spectra in $CDCl_3$

a

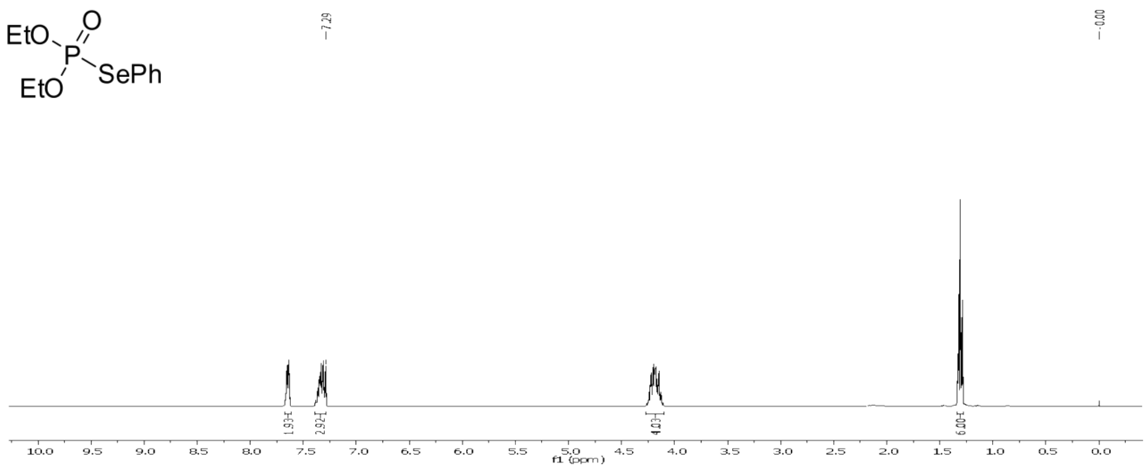

b

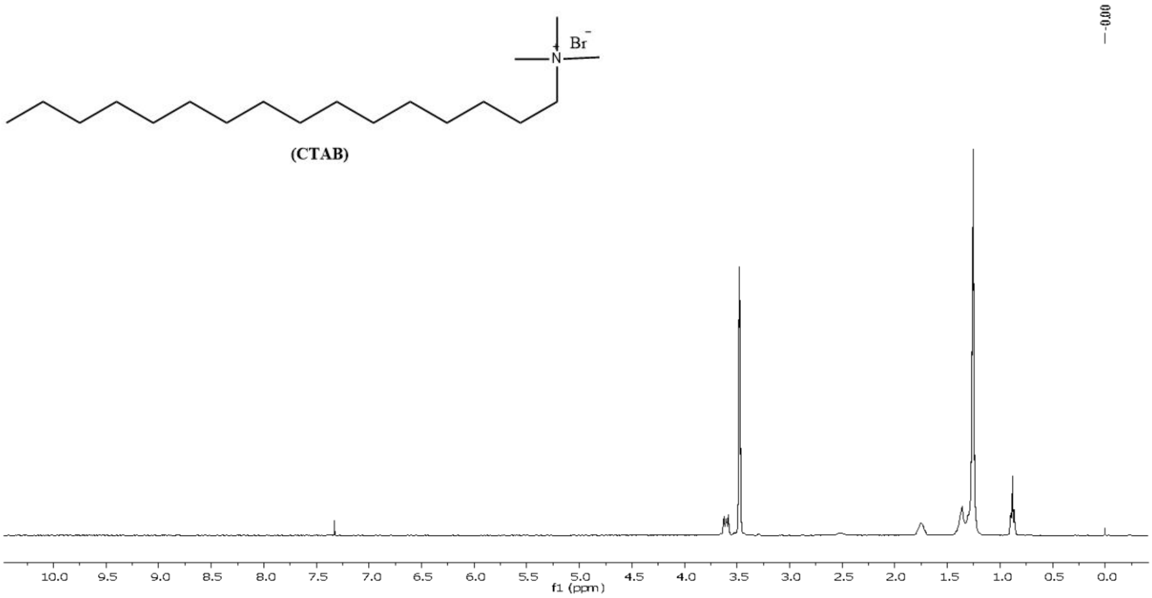

c

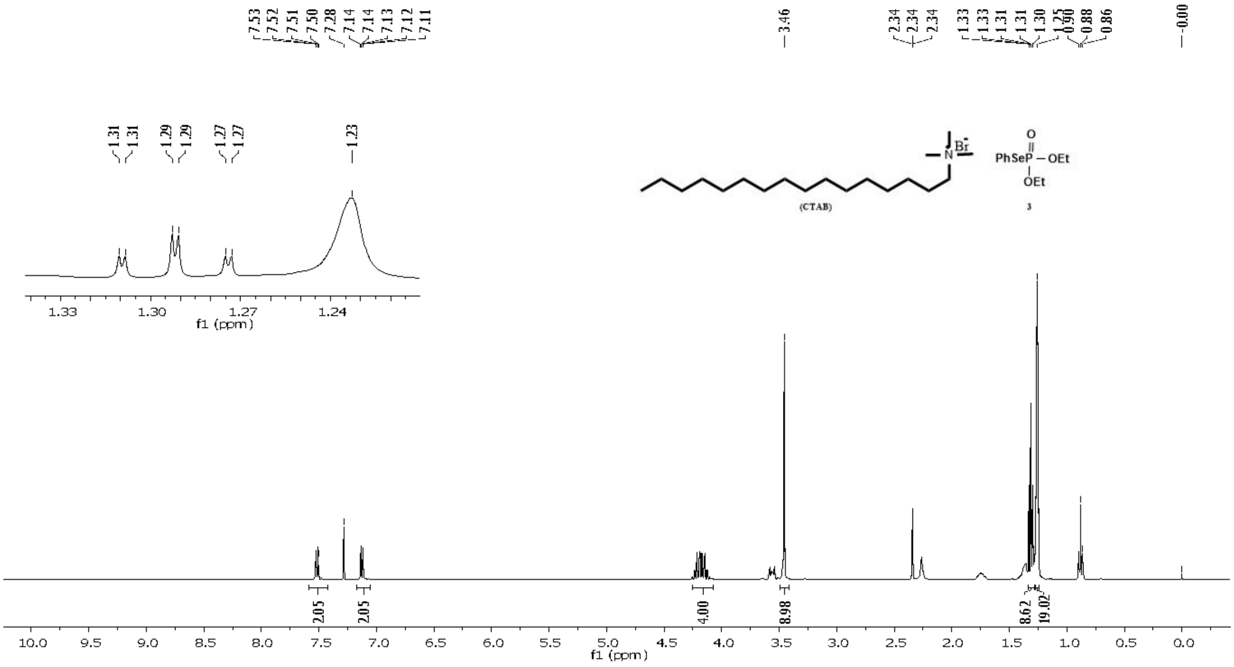
7.53
7.52
7.51
7.50
7.28
7.14
7.14
7.13
7.12
7.11
3.46
2.34
2.34
2.34
1.33
1.33
1.31
1.31
1.30
1.25
0.90
0.88
0.86
0.00
1.31
1.31
1.29
1.29
1.27
1.27
1.23
1.33
1.30
1.27
1.24
f1 (ppm)
Br
N
(CTAB)
O
PhSeP – OEt
OEt
3
2.05
2.05
4.00
8.98
8.62
19.02
10.0 9.5 9.0 8.5 8.0 7.5 7.0 6.5 6.0 5.5 5.0 4.5 4.0 3.5 3.0 2.5 2.0 1.5 1.0 0.5 0.0
f1 (ppm)

d

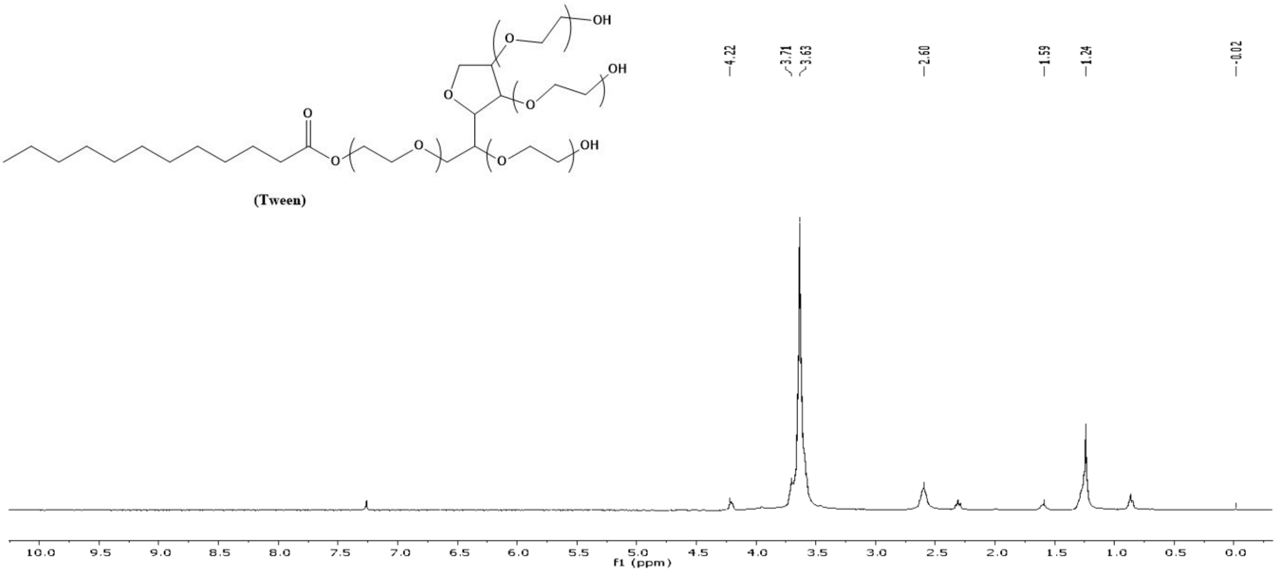
OH
OH
OH
O
O
O
O
O
O
(Tween)
4.22
3.71
3.63
2.60
1.59
1.24
0.02
10.0 9.5 9.0 8.5 8.0 7.5 7.0 6.5 6.0 5.5 5.0 4.5 4.0 3.5 3.0 2.5 2.0 1.5 1.0 0.5 0.0
f1 (ppm)

e

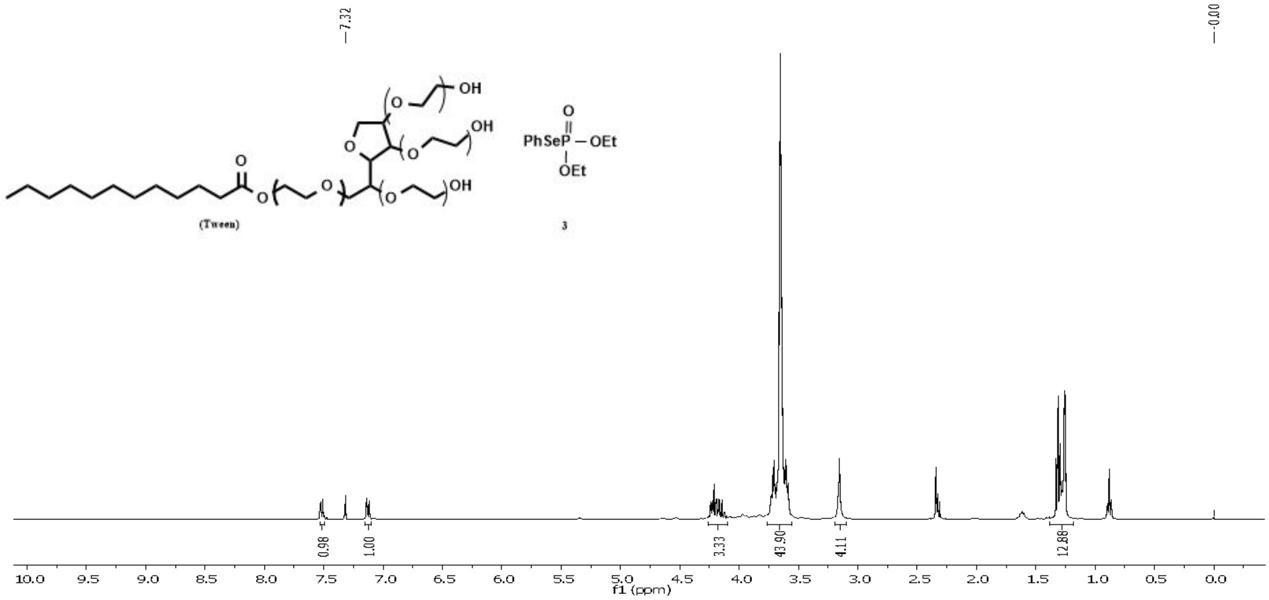

7.32
0.00
(Tween)
PhSeP — OEt
OEt
3
0.98
1.00
3.33
43.90
4.11
12.88
10.0 9.5 9.0 8.5 8.0 7.5 7.0 6.5 6.0 5.5 5.0 4.5 4.0 3.5 3.0 2.5 2.0 1.5 1.0 0.5 0.0
f1 (ppm)

f

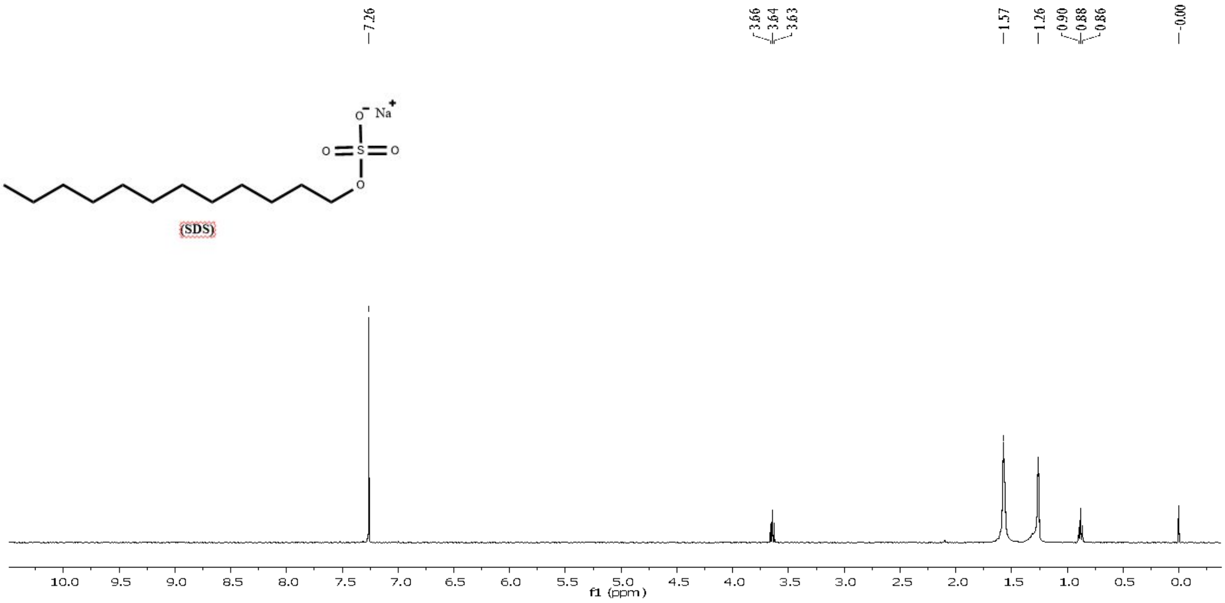

7.26
3.66
3.64
3.63
1.57
1.26
0.90
0.88
0.86
0.00
Na+
(SDS)
10.0 9.5 9.0 8.5 8.0 7.5 7.0 6.5 6.0 5.5 5.0 4.5 4.0 3.5 3.0 2.5 2.0 1.5 1.0 0.5 0.0
f1 (ppm)

g

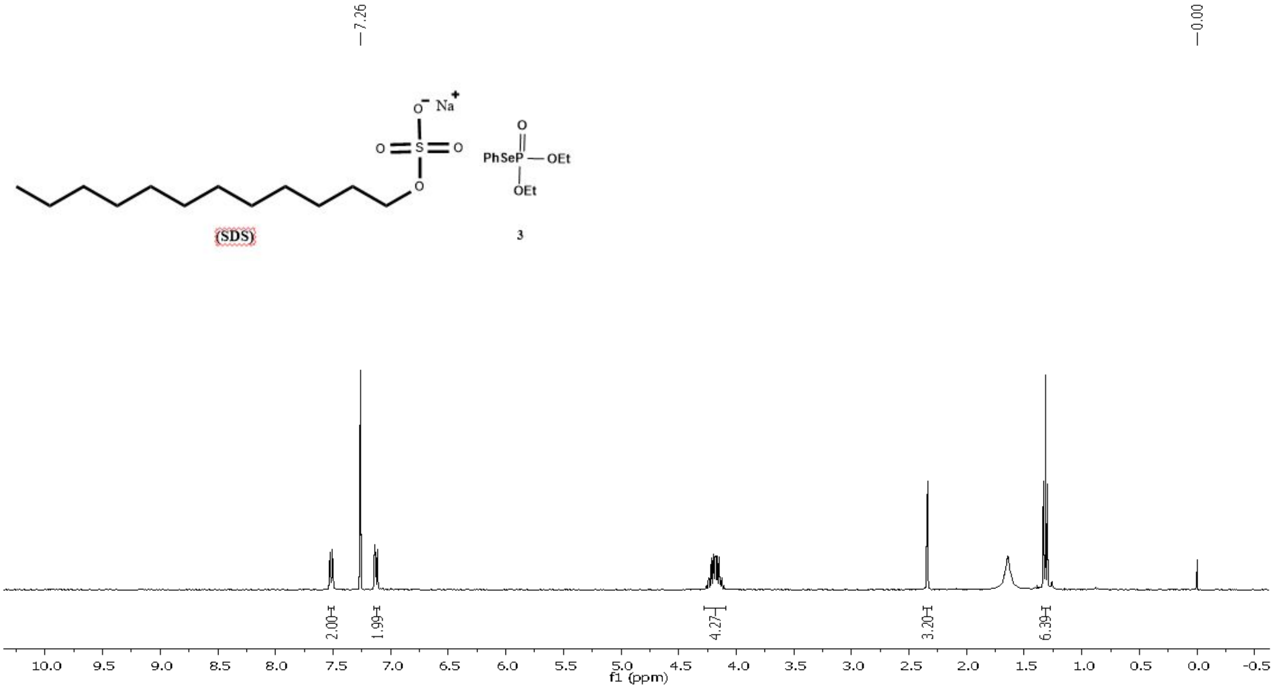

7.26
0.00
(SDS)
PhSeP — OEt
OEt
3
2.00
1.99
4.27
3.20
6.39
10.0 9.5 9.0 8.5 8.0 7.5 7.0 6.5 6.0 5.5 5.0 4.5 4.0 3.5 3.0 2.5 2.0 1.5 1.0 0.5 0.0 -0.5
f1 (ppm)

h

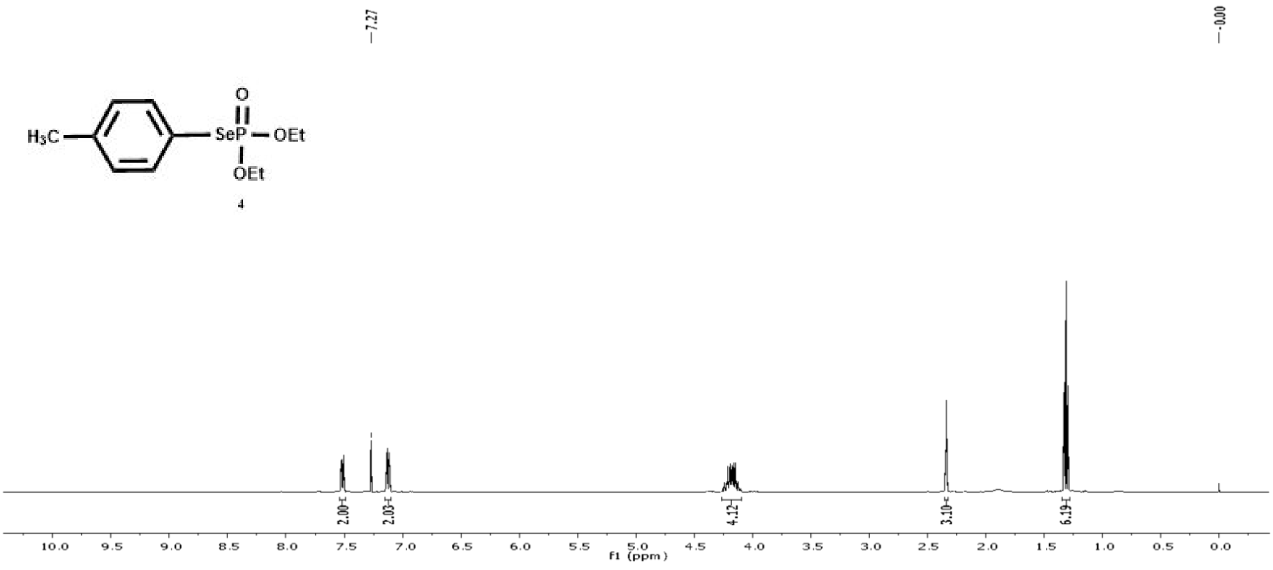

7.27
0.00
H3C
SeP
OEt
OEt
4
2.00
2.03
4.12
3.10
6.19
10.0 9.5 9.0 8.5 8.0 7.5 7.0 6.5 6.0 5.5 5.0 4.5 4.0 3.5 3.0 2.5 2.0 1.5 1.0 0.5 0.0
f1 (ppm)

i

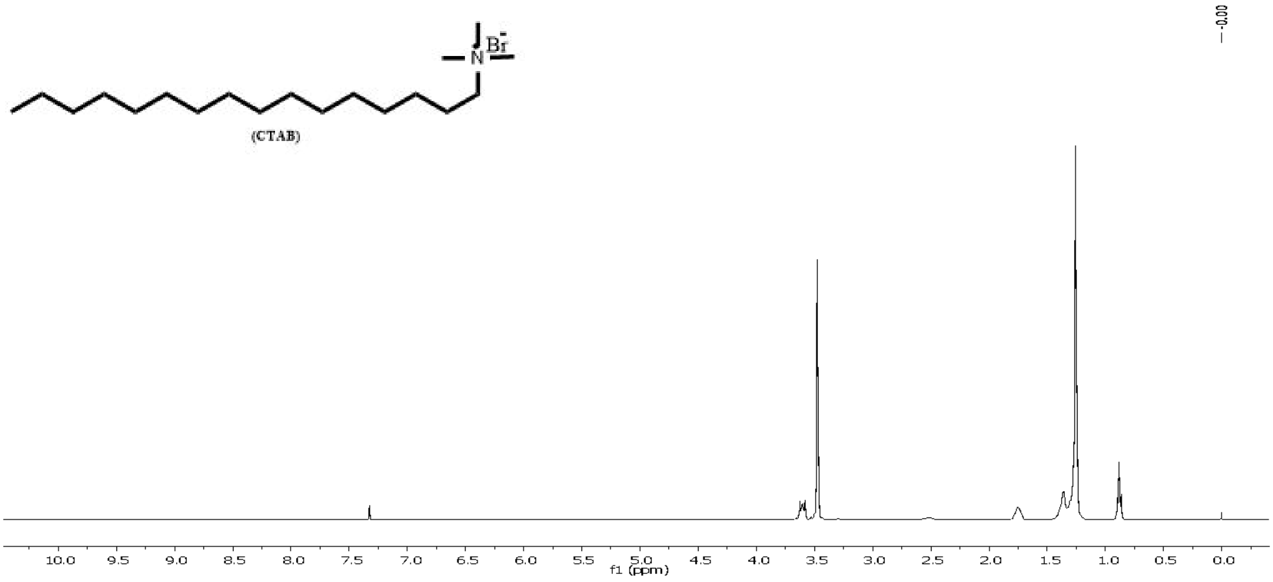

-0.00
Br
N
(CTAB)
10.0 9.5 9.0 8.5 8.0 7.5 7.0 6.5 6.0 5.5 5.0 4.5 4.0 3.5 3.0 2.5 2.0 1.5 1.0 0.5 0.0
f1 (ppm)

j

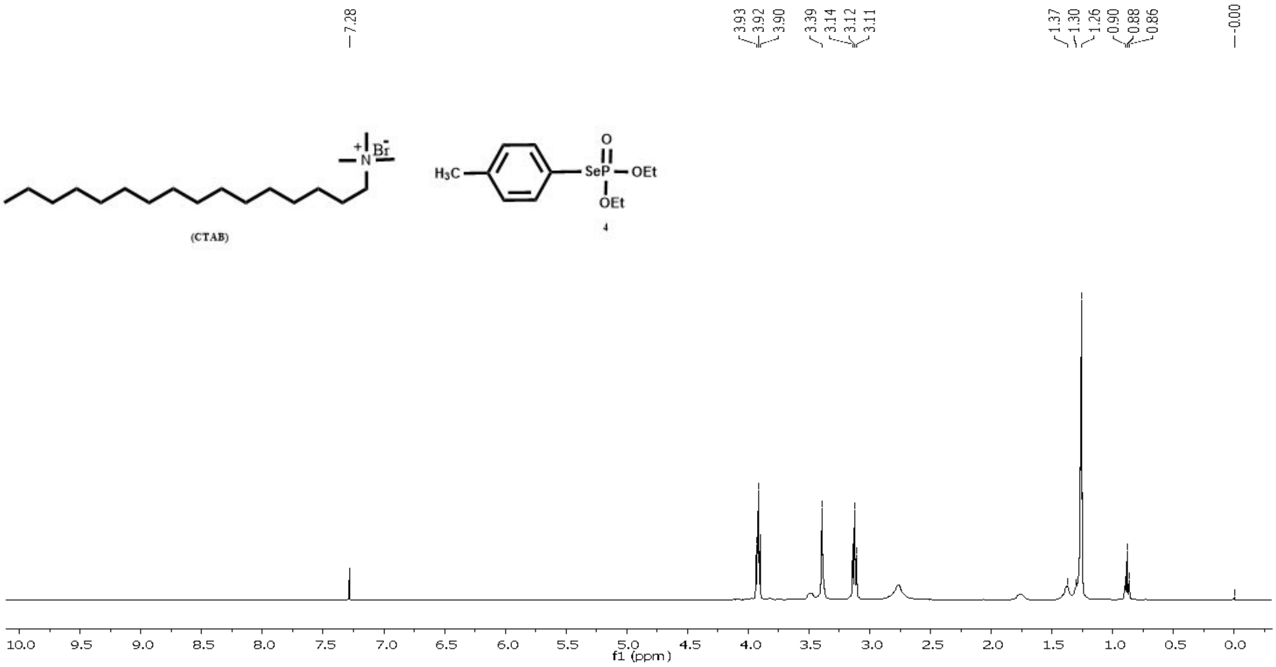

7.28
3.93
3.92
3.90
3.39
3.14
3.12
3.11
1.37
1.30
1.26
0.90
0.88
0.86
0.00
Br
N
(CTAB)
H3C
SeP
O
OEt
OEt
4
10.0 9.5 9.0 8.5 8.0 7.5 7.0 6.5 6.0 5.5 5.0 4.5 4.0 3.5 3.0 2.5 2.0 1.5 1.0 0.5 0.0
f1 (ppm)

k

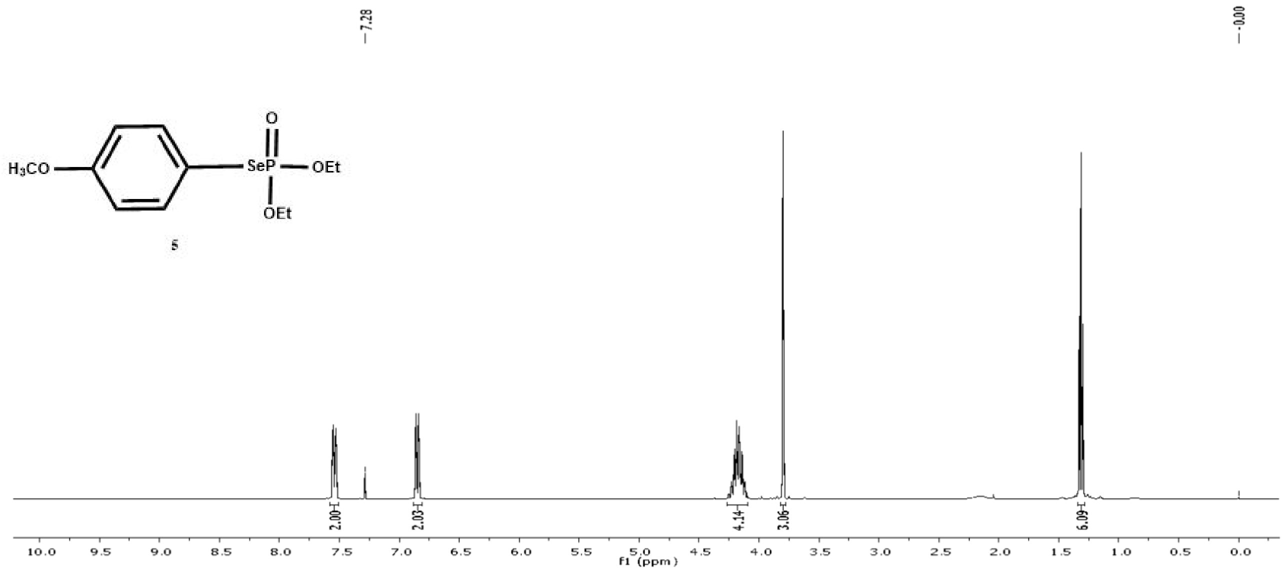

7.28
0.00
O
H_3CO
SeP
OEt
OEt
5
2.00
2.03
4.14
3.06
6.09
10.0 9.5 9.0 8.5 8.0 7.5 7.0 6.5 6.0 5.5 5.0 4.5 4.0 3.5 3.0 2.5 2.0 1.5 1.0 0.5 0.0
f1 (ppm)

l

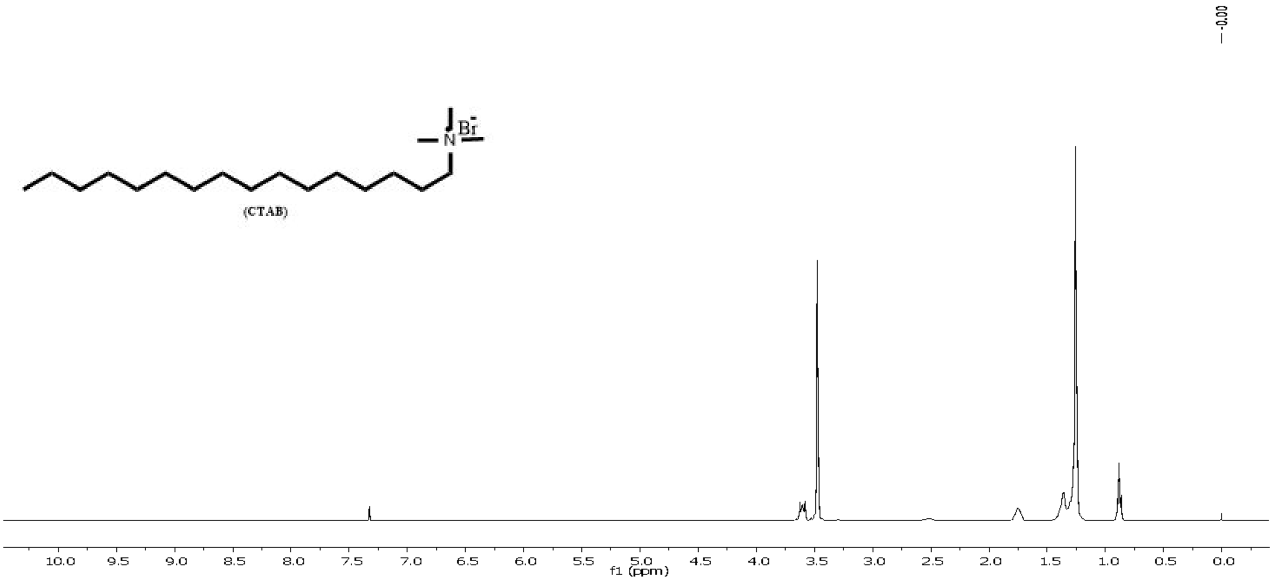

-0.00
N
Br
(CTAB)
10.0 9.5 9.0 8.5 8.0 7.5 7.0 6.5 6.0 5.5 5.0 4.5 4.0 3.5 3.0 2.5 2.0 1.5 1.0 0.5 0.0
f1 (ppm)

m

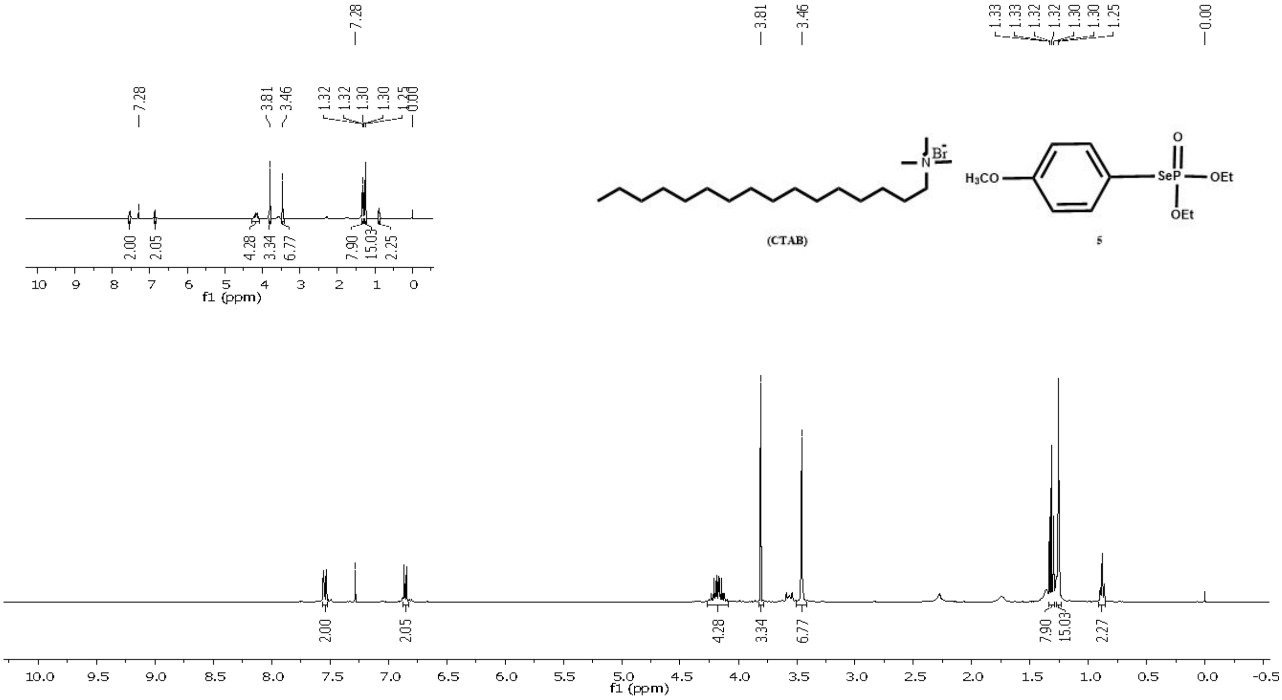

n

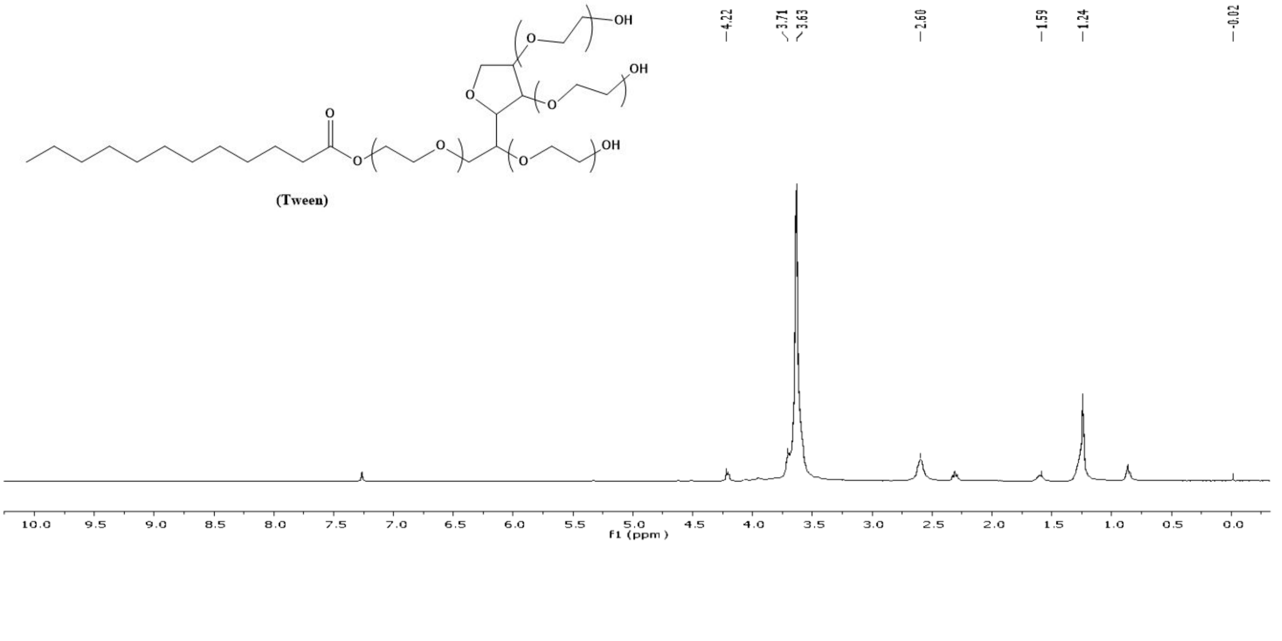

o

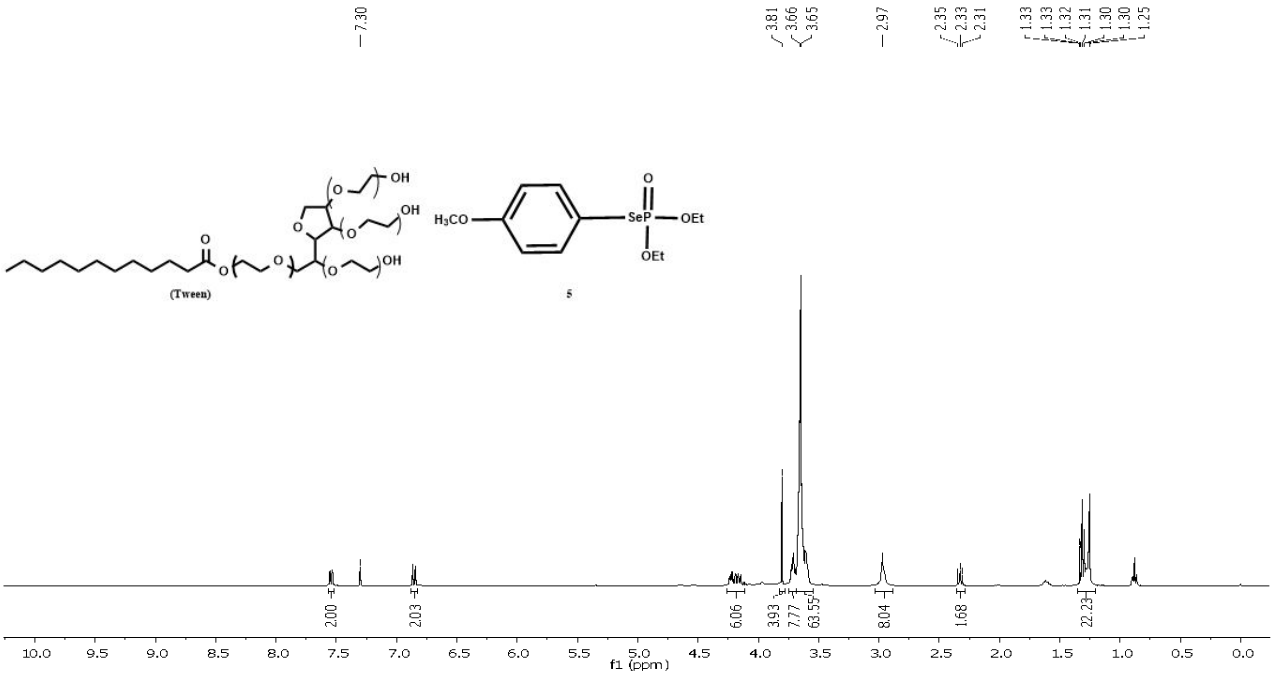

7.30
3.81
3.66
3.65
2.97
2.35
2.33
2.31
1.33
1.33
1.32
1.31
1.30
1.30
1.25
OH
OH
OH
O
(Tween)
H3CO
SeP
OEt
OEt
5
2.00
2.03
6.06
3.93
7.77
63.55
8.04
1.68
22.23
f1 (ppm)

p

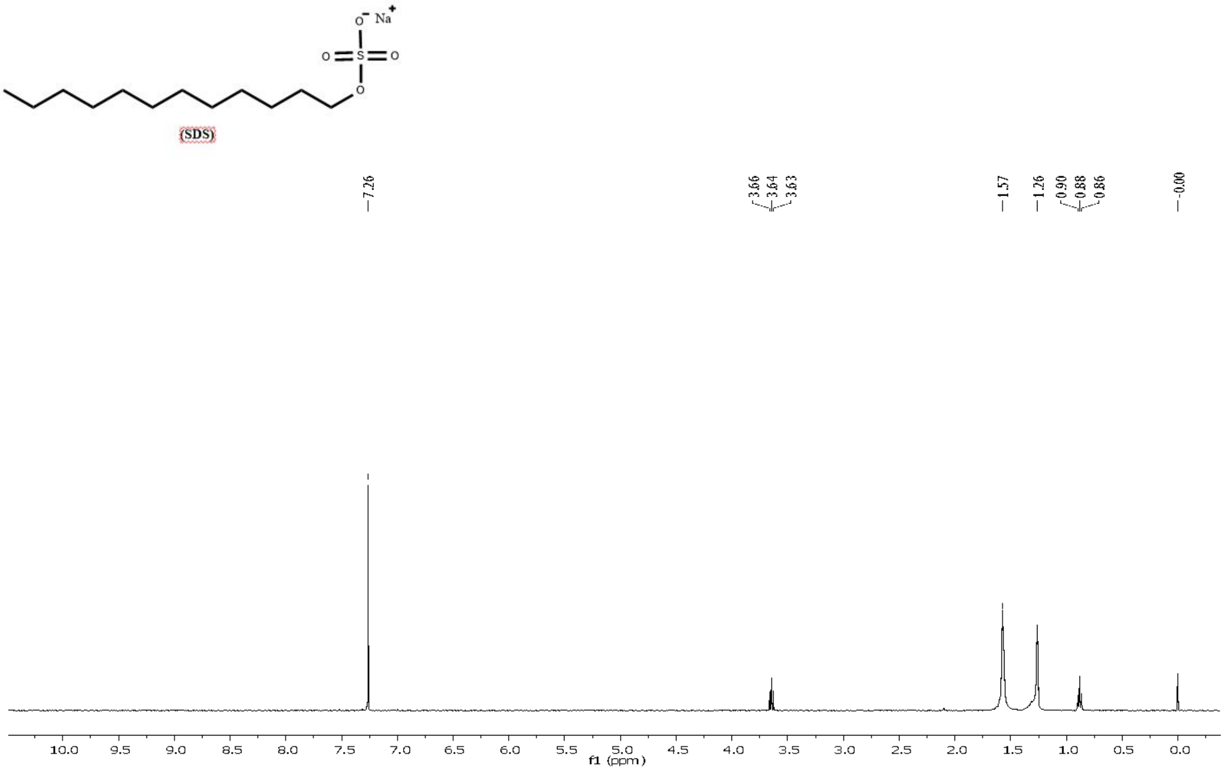

Na+
(SDS)
7.26
3.66
3.64
3.63
1.57
1.26
0.90
0.88
0.86
-0.00
f1 (ppm)

q

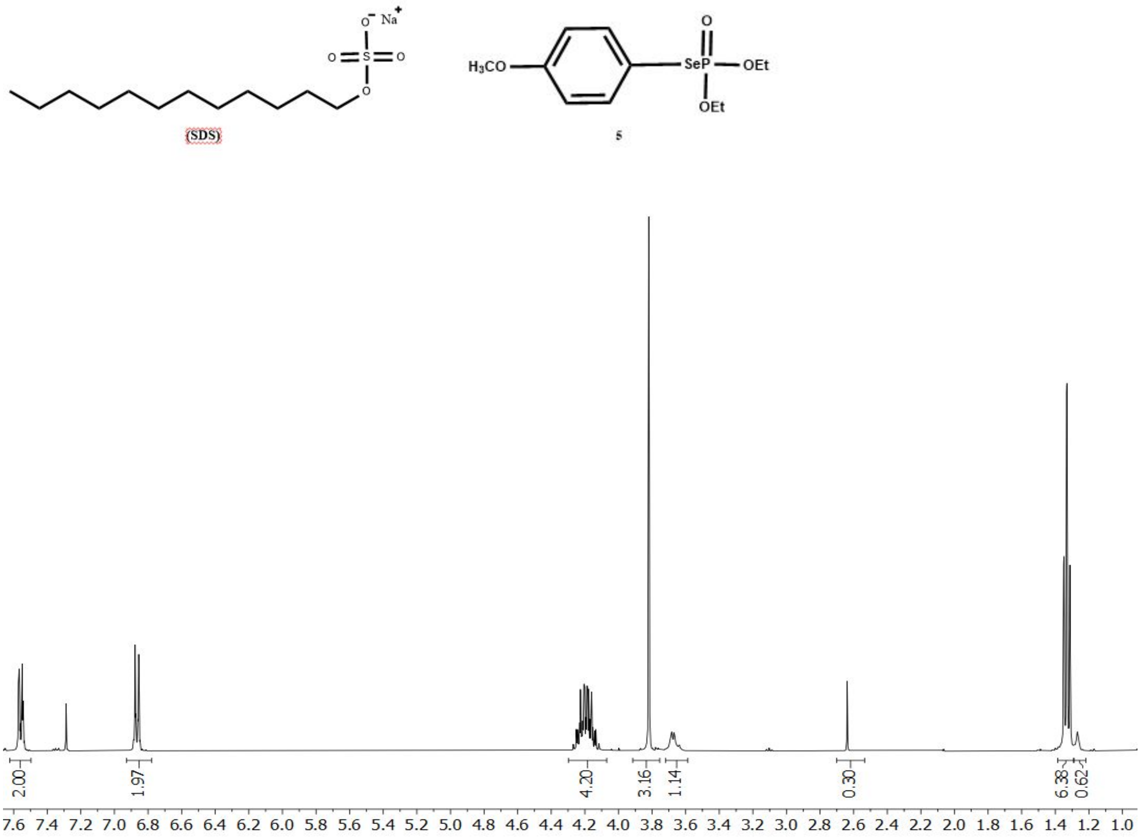

Figure S7. ^{1}H NMR comparative spectra for: a) the Se-phenyl phosphorus selenide (C3); b,i,l) CTAB; c) the Se-phenyl phosphorus selenide (C3) with CTAB; d,n) Tween 20; e) the Se-phenyl phosphorus selenide (C3) with Tween; f,p) SDS; g) the Se-phenyl phosphorus selenide (C3) with SDS; h) diethyl Se-*p*-tolyl phosphorus selenide (C2); j) diethyl Se-*p*-tolyl phosphorus selenide (C2) with CTAB; k) diethyl Se-(4-methoxyphenyl) phosphorus selenide (C1); m) diethyl Se-(4-methoxyphenyl) phosphorus selenide (C1) with CTAB; (o) diethyl Se-(4-methoxyphenyl) phosphorus selenide (C1) with Tween 20; (q) diethyl Se-(4-methoxyphenyl) phosphorus selenide (C1) with SDS.
